# Comparative Transcriptomic Analysis Reveals the Immunosuppressive Targets of Mesalazine in Dextran Sulfate Sodium-Induced Ulcerative Colitis

**DOI:** 10.3389/fgene.2021.698983

**Published:** 2021-08-11

**Authors:** Rong Li, Lin Cheng, Qi Wang, Liming Zhou

**Affiliations:** Department of Pharmacology, West China School of Basic Medical Sciences and Forensic Medicine, Sichuan University, Chengdu, China

**Keywords:** ulcerative colitis, mesalazine, transcriptomics, differentially expressed genes, pharmacological targets

## Abstract

Ulcerative colitis (UC) is a complex inflammatory bowel disorder that can induce colonic and rectal dysfunction. Mesalazine, a first-line medicine, is routinely prescribed for UC treatment. However, the pharmacological targets of mesalazine against UC are not detailed in current publications. In the current study, a transcriptomics strategy was applied to reveal the therapeutic targets and molecular mechanisms of mesalazine for treating dextran sulfate sodium (DSS)-induced UC in mice. Compared with the UC group, a total of 1,663 differentially expressed genes were identified in mesalazine-treated mice, of which 262 were upregulated and 1,401 were downregulated. GO and KEGG enrichment analyses indicated that the protective actions of mesalazine for treating UC were related to the functional regulation of immune inflammatory response, such as the regulation of T cells, white blood cells, and cytokine receptor pathways. In addition, ingenuity pathway analysis of the gene network further revealed the inhibitory action of mesalazine on C–C motif chemokine ligands (CCL11 and CCL21) and C–X–C motif chemokine ligands (CXCL3 and CXCR2). Taken together, the current transcriptomic findings revealed anti-UC pharmacological targets, including the newly discovered biotargets CCL11, CCL21, CXCL3, and CXCR2, of mesalazine against DSS-induced intestinal inflammation.

## Introduction

Ulcerative colitis (UC), a refractory enteritis disease, is characterized by mucosal inflammation and enterocyte lesions present in the gastrointestinal tract (Guan, 2019). It is reported that the prevalence and burden of UC are increasing worldwide, including in China (Ordás et al., 2012). Genetic factors, environmental factors, and dietetic alterations may be crucial risk indicators for UC (Eisenstein, 2018). If left untreated, chronic UC in Asian patients may result in an elevated risk of developing colorectal cancer when compared with that of the general population (Bopanna et al., 2017). The pathological etiology of UC is multifaceted, including impaired barrier function in the mucus layer, lamina propria lesion, gut microbiota imbalance, and neutrophilic immune response (Kobayashi et al., 2020). Early and precise diagnosis of UC using endoscopy, CT scan, blood biomarker determination, fecal calprotectin testing, and bowel ultrasonography is critical to efficacious therapy (Conrad et al., 2014). Treat-to-target, a countermeasure used for treating chronic disorders, may be achieved by determining pathological targets and mitigating UC (Ungaro et al., 2019). Mesalazine, a precursor of 5-aminosalicylic acid, can be used clinically for mild to moderate UC, and is characterized by a good safety profile and low tolerance (Sehgal et al., 2018). Mesalazine is clinically used a the first-line treatment for patients with UC based on its high effectiveness and safety (Kato et al., 2018). However, or new mesalazine anti-UC targets remain minimally reported in preclinical studies. Transcriptomics, a next-generation sequencing technology, is an emerging and rapid method that can identify genome-wide annotation of DNA functions and perform detailed comparative genomic studies in humans and mice (Breschi et al., 2017). Interestingly, transcriptomic strategies have been used to unravel the detailed targets of pathological liver cancer (Lai et al., 2020), perfluorooctanesulfonate-induced immunotoxicity (Li et al., 2020a), glutathione against cleft lip (Li et al., 2021b), and vitamin C against hepatotoxicity (Li et al., 2021a). Therefore, in the present study, we utilized transcriptomic analysis to reveal detailed and novel biotargets of mesalazine against UC *in vivo*.

## Materials and Methods

### Animal Maintenance and Treatment

Mature male C57BL/6J mice, aged around 7 weeks, were purchased from STA Lab Animal Co., Ltd. (Changsha, China). All mice were adaptively maintained for approximately 7 days, and then 3% DSS solution was provided freely in drinking water for UC induction. Meanwhile, DSS-exposed mice were treated with 0.4 g/kg mesalazine for 10 days. In addition, mice treated with DSS-free solution were used as the control group. At the end of the experiment, all mice were euthanized via cervical dislocation, and colorectal tissue samples were immediately isolated and snap-frozen with liquid nitrogen before being used for transcriptomics analysis (Su et al., 2019).

### Transcriptome Sequencing

Total RNA (*n* = 5 from each treatment) was extracted using TRIzol reagent, following the manufacturer’s instructions. RNA samples with a RIN number >8.0, were used for library construction. Briefly, poly(A)-mRNA was enriched from ten-micrograms of total RNA using poly T oligo-attached magnetic beads. After purification, the mRNA was fragmented and subjected to cDNA library construction using an Illumina mRNA Seq sample preparation kit. Paired-end sequencing (150 bp each) was performed on an Illumina HiSeq 4000, following the manufacturer’s instructions.

### Bioinformatic Analysis of Transcriptome

The adaptor sequences and sequencing primers were trimmed. Low-quality reads with *q* quality scores lower than 20 were removed. The clean sequence reads were mapped to the UCSC reference genome^1^ using the HISAT package. StringTie and edgeR were used to determine the expression levels of all the transcripts. Genes with a lLog_2_ (fold change) > 1 and with statistical significance (*q*-value < 0.05) were defined as differentially expressed genes (DEGs). The DEGs were subjected to Gene Ontology (GO), Kyoto Encyclopedia of Genes and Genomes (KEGG) enrichment analysis, and ingenuity pathway analysis (IPA) to examine the effects of mesalazine on DSS-induced colitis.

### Enzyme-Linked Immunosorbent Assay

The colon samples were homogenized, and the supernatants were used for the measurement of inflammatory cytokines using ELISA kits (Elisa Biotech, Shanghai, China). The experimental procedures were performed as described previously (Xu et al., 2019).

### Immunostaining

The colon samples from different groups were fixed with 4% paraformaldehyde prior to being cut into 4–5 μm thick sections using a microtome. Thereafter, the sections were dewaxed using xylene and were stained with hematoxylin and eosin dyes for histopathological examination (Wu et al., 2019; Li et al., 2020b).

## Discussion

In this study, we performed comparative transcriptomic analysis to understand the molecular basis of DSS-induced colitis and to delineate the molecular mechanism underlying the immunosuppressive effect of mesalazine. In the first part of the study, we attempted to understand the detailed mechanism underlying DSS-induced colitis in a mouse model. It has been well reported that the major cause of UC results from abnormal immune responses to antigens derived from the intestinal microbiota (Podolsky, 2002; Macdonald and Monteleone, 2005). Our results showed that DSS could lead to a large number of differentially expressed genes. The results of GO and pathway analyses further highlighted that the induction of inflammatory and immune responses is mainly through alterations of cytokine-cytokine receptor interaction, IL-17 signaling pathway, leukocyte transendothelial migration, Toll-like receptor signaling pathway, and intestinal immune network for IgA production. In addition, IBD was highlighted in the analysis, suggesting that the model was valid. Cytokine-cytokine receptor interactions play an important role in immune regulation (Wu et al., 2005). It is involved in the T-cell receptor signaling pathway (Qian et al., 2019) and has been reported to be associated with autoimmune disorders (Liu et al., 2013; Xing et al., 2020). Many autoimmune and inflammatory diseases can result from excessive cytokine production or responsiveness. The IL-17 signaling pathway is the founding member of a novel family of inflammatory cytokines (Amatya et al., 2017). IL-17, the hallmark cytokine of the newly defined T helper 17 [T(H)17] cell subset, plays a crucial role in the inflammatory pathology of autoimmune diseases (Gaffen, 2009). The role of the IL-17 family in most common autoimmune diseases, such as psoriasis, IBD, and rheumatoid arthritis, has been extensively characterized (Zhang et al., 2015). Our funding is concordant with the previous IL-17R knockout mice study that IL-17 signaling plays a critical role in the development of TNBS-induced colitis and may represent a target for therapeutic intervention for IBD (Zhang et al., 2006). The toll-like receptor signaling pathway has been recognized as a key repressor of inflammatory signaling and plays an essential role in the pathogenesis of autoimmune diseases (Chen et al., 2016; Shamilov and Aneskievich, 2018). It is a primary sensor of both innate and adaptive immune systems through its modulation of numerous genes that function in host defense, including inflammatory cytokines, chemokines, and antigen presenting molecules (Liu et al., 2006). It has been reported that Toll-like receptor (TLR) signaling-related protein (TLR4) was detected in the colon tissue of DSS model mice (Bai et al., 2021). Furthermore, macrophages in the intestinal mucosa can rapidly induce TLR-mediated inflammatory responses (Wang et al., 2021). Our results provide a better understanding of the immunopathogenesis of UC and may help to identify novel targets for more potent interventions.

## Results

### DSS-Induced Colitis Through the Induction of Inflammatory Response

**FIGURE 1:**
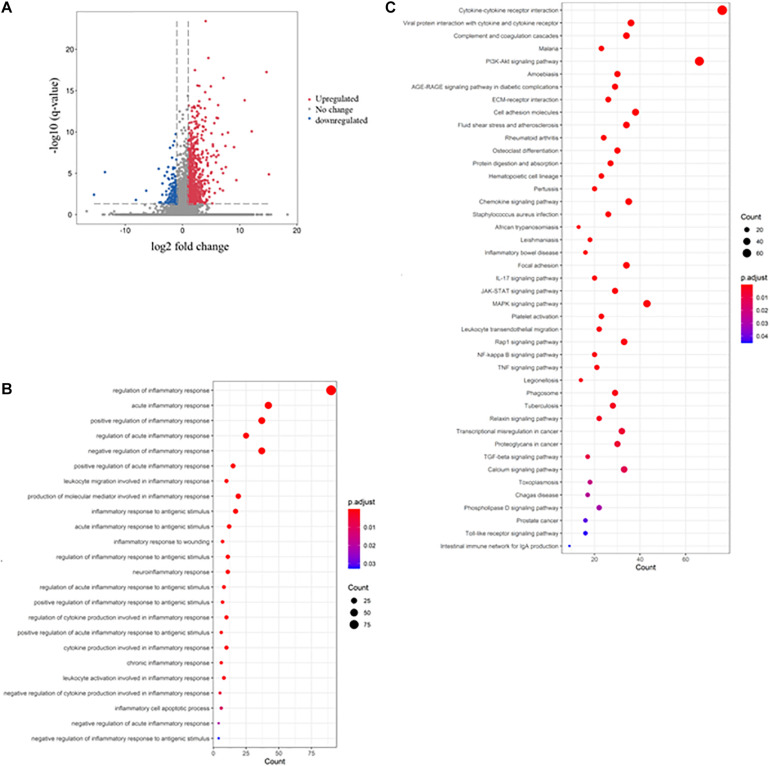
Dextran sulfate sodium (DSS) induced colitis through the induction of inflammatory response. The volcano plot shows the differential expression of genes in the gut of DSS-induced colitis mice. The genes with | log_2_ (fold change: DSS/Ctrl)| > 1 and -log B and H corrected *p*-value > 1.3 were considered differentially expressed genes (DEGs). The red dot represents upregulated genes, the green dot represents downregulated genes, and the gray dot represents genes with no significant change. **(B)** The rich factor plot shows the alteration of biological processes related to immunity in the gut of DSS-induced colitis mice. The size of the dot represents the number of DEGs. The color intensity of the dots represents the significance of the biology processes. **(C)** The rich factor plot showed the alteration of cell signaling pathways related to immunity in the gut of DSS-induced colitis mice. The size of the dot represents the number of DEGs. The color intensity of the dots represents the significance of the signaling pathways.

To understand the molecular mechanism underlying DSS-induced colitis and the immunosuppressive effects of mesalazine on DSS-induced colitis, a comparative transcriptomic analysis was conducted, followed by bioinformatic analysis. Deep sequencing of RNA libraries derived from control and treatment groups each generated at least 43 million quality-trimmed clean reads (Supplementary Table 1); the read utilization ratio was over 97%. A total of 110 Gb quality-trimmed bases were obtained from transcriptome sequencing (Supplementary Table 1). Over 90% of the sequencing reads could be mapped to the exonic regions of the mouse reference genome (Supplementary Table 2). In the comparative transcriptomic analysis, a total of 1,627 differentially expressed genes (DEGs), including 1,326 upregulated and 301 downregulated genes, were identified in the DSS-induced colitis mice as compared to the control group mice (Figure 1A and Supplementary Table 3). The DEGs were then subjected to GO and KEGG enrichment analyses to understand the alteration of biological functions and signaling pathways in the DSS-induced colitis model. GO results suggested that numerous biological processes, cellular components, and molecular functions were altered in the DSS-induced colitis model. In the analysis of biological processes, we found the activation of numerous inflammatory processes (Figure 1B and Table 1). The KEGG pathway analysis further highlighted the alteration of pathways related to immune and inflammatory responses, including cytokine-cytokine receptor interaction, inflammatory bowel disease (IBD), IL-17 signaling pathway, leukocyte transendothelial migration, Toll-like receptor signaling pathway, and intestinal immune network for IgA production (Figure 1C and Table 2).

**TABLE 1:** Inflammatory responses in gut caused by DSS.

GO ID	Description	Adjusted *p*-value	Number of gene	Gene ID
GO:0050727	regulation of inflammatory response	5.65E-32	90	S100a8/S100a9/Il1rl1/Il6/Mmp8/Serpine1/Il1b/Ctla2a/Ptgs2/Il1r2/Apod/Il1r1/Sema7a/C3/Fcgr3/Per1/Osm/Fcgr2b/Cdh5/Tnfsf11/Ggt1/Fcer1g/Il33/Tgm2/Nlrp3/Foxf1/Ednra/Ier3/Tarm1/Cd200/Tnf/Aoah/Alox5ap/Fcgr1/Calcrl/Ccl3/Acod1/Serping1/Siglece/Adam8/Socs3/Zc3h12a/Ets1/Gprc5b/Cfh/Mefv/Ager/Nos2/Apoe/Fabp4/Ednrb/A2m/Tac1/Foxp3/Wnt5a/Il10/Il2ra/Sirpa/Cd55/Tnfaip6/Gpx2/Ccr7/Ccr2/Trpv4/Apoa1/Rora/Bst1/Ffar4/Tlr2/Ccr5/Adora1/Gata3/Hgf/Lbp/Pbk/Adamts12/Gpsm3/Adora2a/Cnr1/Tnfaip8l2/Fanca/Agt/Clcf1/Slc7a2/Cd6/Il22/Slamf8/Pik3ap1/C6/Il20rb
GO:0002526	acute inflammatory response	4.54E-19	42	Il6/Serpina3n/Cxcr2/Saa3/Il1b/Ptgs2/Hp/Cxcl1/Orm2/Trem1/C3/Fcgr3/Fcgr2b/Il1a/Tnfsf11/Fcer1g/Nlrp3/Tnf/Alox5ap/Vnn1/Fcgr1/Serping1/Cd163/Adam8/Fn1/Cfh/Ednrb/A2m/Tac1/Nupr1/Icam1/Cd55/Bdkrb2/Ccr7/Ccr5/Adora1/Lbp/Cnr1/F3/Cd6/C6/Il20rb
GO:0050729	positive regulation of inflammatory response	6.22E-14	37	S100a8/S100a9/Il1rl1/Il6/Mmp8/Serpine1/Il1b/Ptgs2/C3/Fcgr3/Osm/Tnfsf11/Fcer1g/Il33/Tgm2/Ednra/Tarm1/Tnf/Alox5ap/Fcgr1/Ccl3/Adam8/Ets1/Gprc5b/Ager/Fabp4/Tac1/Wnt5a/Ccr7/Ccr2/Trpv4/Tlr2/Ccr5/Lbp/Gpsm3/Cnr1/Cd6
GO:0002673	regulation of acute inflammatory response	6.08E-13	25	Il6/Il1b/Ptgs2/C3/Fcgr3/Fcgr2b/Tnfsf11/Fcer1g/Nlrp3/Tnf/Alox5ap/Fcgr1/Serping1/Adam8/Cfh/Ednrb/A2m/Tac1/Cd55/Ccr7/Ccr5/Adora1/Cnr1/C6/Il20rb
GO:0050728	negative regulation of inflammatory response	1.11E-12	37	Ctla2a/Il1r2/Apod/Fcgr2b/Cdh5/Nlrp3/Foxf1/Ier3/Cd200/Aoah/Calcrl/Acod1/Siglece/Socs3/Zc3h12a/Ets1/Mefv/Ager/Apoe/Foxp3/Il10/Il2ra/Sirpa/Tnfaip6/Gpx2/Apoa1/Rora/Ffar4/Adora1/Gata3/Hgf/Pbk/Adora2a/Tnfaip8l2/Il22/Slamf8/Il20rb
GO:0002675	positive regulation of acute inflammatory response	1.15E-08	15	Il6/Il1b/Ptgs2/C3/Fcgr3/Tnfsf11/Fcer1g/Tnf/Alox5ap/Fcgr1/Adam8/Tac1/Ccr7/Ccr5/Cnr1
GO:0002523	leukocyte migration involved in inflammatory response	1.92E-07	10	S100a8/S100a9/Ccl2/Itgam/Ppbp/Adam8/Itgb2/Lbp/Slamf8/Jam3
GO:0002532	production of molecular mediator involved in inflammatory response	5.11E-07	19	Serpine1/Il1r2/Apod/Per1/Fcer1g/Tarm1/Alox5ap/Zc3h12a/Mefv/Nos2/Cd300a/Il4ra/Sirpa/Lbp/Gpsm3/P2rx1/Slc7a2/Cd6/Slamf8
GO:0002437	inflammatory response to antigenic stimulus	7.40E-07	17	Cxcr2/C3/Fcgr3/Fcgr2b/Fcer1g/Tnf/Fcgr1/Il5ra/Il10/Icam1/Il2ra/Bdkrb2/Gpx2/Ccr7/Cnr1/Cd6/Il20rb
GO:0002438	acute inflammatory response to antigenic stimulus	8.96E-07	12	Cxcr2/C3/Fcgr3/Fcgr2b/Fcer1g/Fcgr1/Icam1/Bdkrb2/Ccr7/Cnr1/Cd6/Il20rb
GO:0090594	inflammatory response to wounding	1.01E-05	7	Timp1/Hmox1/Il1a/Ager/Clec10a/F2r/Ccr2
GO:0002861	regulation of inflammatory response to antigenic stimulus	3.62E-05	11	C3/Fcgr3/Fcgr2b/Fcer1g/Tnf/Fcgr1/Il10/Gpx2/Ccr7/Cnr1/Il20rb
GO:0150076	neuroinflammatory response	0.000105408	11	Mmp8/Fpr2/Il33/Cd200/Ager/Tlr2/Tlr8/Tlr7/Adora2a/Agt/Aif1
GO:0002864	regulation of acute inflammatory response to antigenic stimulus	0.000161112	8	C3/Fcgr3/Fcgr2b/Fcer1g/Fcgr1/Ccr7/Cnr1/Il20rb
GO:0002863	positive regulation of inflammatory response to antigenic stimulus	0.000577499	7	C3/Fcgr3/Fcer1g/Tnf/Fcgr1/Ccr7/Cnr1
GO:1900015	regulation of cytokine production involved in inflammatory response	0.000633959	10	Il1r2/Apod/Per1/Tarm1/Zc3h12a/Mefv/Nos2/Sirpa/Gpsm3/Cd6
GO:0002866	positive regulation of acute inflammatory response to antigenic stimulus	0.00098818	6	C3/Fcgr3/Fcer1g/Fcgr1/Ccr7/Cnr1
GO:0002534	cytokine production involved in inflammatory response	0.00109548	10	Il1r2/Apod/Per1/Tarm1/Zc3h12a/Mefv/Nos2/Sirpa/Gpsm3/Cd6
GO:0002544	chronic inflammatory response	0.001408563	6	Gja1/Tnf/Vnn1/Cebpb/Foxp3/Il10
GO:0002269	leukocyte activation involved in inflammatory response	0.001863998	8	Mmp8/Fpr2/Il33/Ager/Tlr2/Tlr8/Tlr7/Aif1
GO:1900016	negative regulation of cytokine production involved in inflammatory response	0.006573282	5	Il1r2/Apod/Zc3h12a/Mefv/Sirpa
GO:0006925	inflammatory cell apoptotic process	0.012571732	6	Il6/Cxcr2/Fcgr2b/Ccr5/Hcar2/Slc7a11
GO:0002674	negative regulation of acute inflammatory response	0.02079512	4	Fcgr2b/Nlrp3/Adora1/Il20rb
GO:0002862	negative regulation of inflammatory response to antigenic stimulus	0.032741163	4	Fcgr2b/Il10/Gpx2/Il20rb

**TABLE 2:** Alterations of cell signaling pathways in gut caused by DSS.

KEGG ID	Description	Adjusted *p*-value	Number of gene	Gene ID
mmu04060	Cytokine-cytokine receptor interaction	1.03E-18	76	Cxcl5/Il1rl1/Il6/Cxcl2/Csf3/Cxcr2/Inhbb/Il11/Il1b/Il19/Cxcl1/Il1r2/Osmr/Il1r1/Cxcl3/Csf3r/Ccl2/Osm/Tnfrsf9/Il1a/Tnfsf11/Il33/Ppbp/Cxcl13/Bmp3/Bmp7/Inhba/Tnf/Ccr1/Ccl11/Il5ra/Ccl3/Csf2rb2/Csf2rb/Pf4/Ccl8/Il18rap/Cxcl12/Bmpr2/Ackr3/Il6st/Tgfb1/Cxcl16/Tgfbr1/Il4ra/Cd4/Ifnar2/Il34/Ccl4/Csf1r/Il10ra/Il10/Tnfrsf11b/Il2ra/Il13ra2/Ccr7/Ccr2/Ccr5/Bmp5/Tnfrsf12a/Gm10591/Cxcr4/Cntfr/Il7r/Gm13304/Clcf1/Il18r1/Bmp8b/Relt/Il22/Tnfsf9/Il2rb/Tnfrsf4/Il20rb/Klk1b4/Ccl21a
mmu04061	Viral protein interaction with cytokine and cytokine receptor	4.83E-14	36	Cxcl5/Il6/Cxcl2/Cxcr2/Il19/Cxcl1/Cxcl3/Ccl2/Ppbp/Cxcl13/Tnf/Ccr1/Ccl11/Ccl3/Pf4/Ccl8/Il18rap/Cxcl12/Ackr3/Il6st/Il34/Ccl4/Csf1r/Il10ra/Il10/Il2ra/Ccr7/Ccr2/Ccr5/Gm10591/Cxcr4/Gm13304/Il18r1/Il2rb/Il20rb/Ccl21a
mmu04610	Complement and coagulation cascades	7.62E-13	34	Serpine1/Plaur/C3/Bdkrb1/Plau/Plat/Fga/F10/C1qa/Itgam/Fgg/C1s1/C4b/F13a1/C1qb/Vwf/Serping1/Cfh/C1qc/C1ra/A2m/Pros1/Itgb2/C7/Cd55/Bdkrb2/F2rl3/F2r/C1rb/Itgax/Thbd/F3/C6/C3ar1
mmu05144	Malaria	1.21E-09	23	Il6/Csf3/Hbb-bs/Thbs1/Hba-a2/Hba-a1/Il1b/Hbb-bt/Ccl2/Selp/Sele/Vcam1/Pecam1/Tnf/Cd36/Tgfb1/Itgb2/Il10/Icam1/Gypc/Tlr2/Hgf/Klrb1b
mmu04151	PI3K-Akt signaling pathway	4.99E-09	66	Il6/Csf3/Thbs1/Ereg/Osmr/Col4a1/Tnc/Csf3r/Col4a2/Lamb1/Osm/Lamc1/Lama1/Pdgfra/Pgf/Creb3l3/Lama4/Nos3/Flt1/Nr4a1/Spp1/Fgf7/Vwf/Itgb8/Fn1/Igf1/Angpt1/Itga5/Fgfr1/Col6a2/Efna5/Phlpp2/Il4ra/Col6a4/Pdgfrb/Pdgfc/Ifnar2/Col6a1/Ddit4/Angpt2/Csf1r/Nras/Il2ra/Gng2/Kdr/Col1a2/Lama5/Myb/F2r/Ppp2r2b/Pdgfb/Col1a1/Fgfr3/Tlr2/Sgk1/Pik3r6/Hgf/Itgb3/Pik3r5/Col4a4/Il7r/Creb5/Pik3ap1/Il2rb/Gng4/Klk1b4
mmu05146	Amoebiasis	2.62E-08	30	Il6/Cxcl2/Il1b/Cxcl1/Il1r2/Il1r1/Col4a1/Cxcl3/Col4a2/Lamb1/Lamc1/Lama1/Itgam/Tnf/Arg2/Lama4/Arg1/Fn1/Hspb1/Nos2/Tgfb1/Itgb2/Il10/Col1a2/Lama5/Col3a1/Col1a1/Tlr2/Serpinb6b/Col4a4
mmu04933	AGE-RAGE signaling pathway in diabetic complications	2.62E-08	29	Il6/Serpine1/Il1b/Col4a1/Ccl2/Col4a2/Sele/Il1a/Vcam1/Mmp2/Tnf/Nos3/Nox1/Fn1/Ager/Egr1/Tgfb1/Tgfbr1/Nras/Icam1/Col1a2/Col3a1/Col1a1/Cybb/Thbd/Col4a4/F3/Agt/Plcd3
mmu04512	ECM-receptor interaction	8.92E-08	26	Thbs1/Col4a1/Tnc/Col4a2/Lamb1/Lamc1/Lama1/Frem1/Lama4/Spp1/Vwf/Itgb8/Fn1/Cd36/Itga5/Col6a2/Col6a4/Col6a1/Col1a2/Lama5/Hmmr/Col1a1/Itgb3/Npnt/Col4a4/Sv2c
mmu04514	Cell adhesion molecules	2.63E-07	38	Selp/Sele/Cdh5/Vcam1/Itgam/Pecam1/Ctla4/Esam/Icos/Vcan/Itgb8/Vsir/Madcam1/Cd4/Cldn4/Nlgn2/Cd80/Itgb2/Icam1/Cd86/Sell/H2-Q10/Selplg/Tigit/Cadm1/Pdcd1/Cldn5/Ntng1/Ntng2/Jam2/Cldn1/Cadm3/Cd6/Siglec1/Jam3/Vtcn1/Cd34/Nfasc
mmu05418	Fluid shear stress and atherosclerosis	3.14E-07	34	Il1b/Il1r2/Il1r1/Mmp9/Ccl2/Dusp1/Sele/Plat/Hmox1/Il1a/Cdh5/Vcam1/Mmp2/Pecam1/Gstm3/Tnf/Nos3/Nox1/Klf2/Bmpr2/Gsta4/Icam1/Kdr/Cav1/Pdgfb/Trpv4/Gstm6/Rac2/Gsta3/Itgb3/Thbd/Gm3776/Gstm7/Ncf1
mmu05323	Rheumatoid arthritis	1.16E-06	24	Cxcl5/Il6/Mmp3/Cxcl2/Il11/Il1b/Cxcl1/Cxcl3/Ccl2/Il1a/Tnfsf11/Tnf/Ctla4/Flt1/Ccl3/Ctsk/Cxcl12/Angpt1/Tgfb1/Cd80/Itgb2/Icam1/Cd86/Tlr2
mmu04380	Osteoclast differentiation	1.38E-06	30	Il1b/Il1r1/Fcgr3/Fcgr2b/Il1a/Tnfsf11/Lilrb4a/Fcgr4/Tnf/Pirb/Fcgr1/Trem2/Tyrobp/Ctsk/Nox1/Socs3/Tgfb1/Tgfbr1/Spi1/Ifnar2/Csf1r/Tnfrsf11b/Sirpa/Itgb3/Lcp2/Sirpb1b/Fyn/Fosl1/Ncf4/Ncf1
mmu04974	Protein digestion and absorption	1.40E-06	27	Col18a1/Cpa3/Col4a1/Col4a2/Gm2663/Kcne3/Col15a1/Col5a2/Atp1b2/Xpnpep2/Col13a1/Col6a2/Eln/Col5a1/Col6a4/Col5a3/Col6a1/Col12a1/Slc36a2/Col23a1/Col1a2/Col3a1/Col1a1/Slc36a4/Col4a4/Col24a1/Col16a1
mmu04640	Hematopoietic cell lineage	1.82E-05	23	Il6/Csf3/Il11/Il1b/Il1r2/Il1r1/Csf3r/Il1a/Itgam/Tnf/Cd33/Fcgr1/Il5ra/Cd36/Itga5/Il4ra/Cd4/Csf1r/Il2ra/Cd55/Itgb3/Il7r/Cd34
mmu05133	Pertussis	3.16E-05	20	Cxcl5/Il6/Il1b/C3/Il1a/C1qa/Itgam/Nlrp3/Tnf/C1s1/C4b/C1qb/Serping1/Itga5/C1qc/Nos2/C1ra/Itgb2/Il10/C1rb
mmu04062	Chemokine signaling pathway	5.47E-05	35	Cxcl5/Cxcl2/Cxcr2/Cxcl1/Cxcl3/Ccl2/Ppbp/Cxcl13/Ccr1/Ccl11/Ccl3/Grk3/Pf4/Ccl8/Cxcl12/Prex1/Arrb2/Cxcl16/Ccl4/Adcy4/Nras/Gng2/Ccr7/Ccr2/Ccr5/Rac2/Pik3r6/Gm10591/Cxcr4/Pik3r5/Gm13304/Gng4/Ncf1/Fgr/Ccl21a
mmu05150	Staphylococcus aureus infection	6.93E-05	26	Fpr2/Fpr1/Selp/C3/Fcgr3/Fcgr2b/C1qa/Itgam/Fgg/Fcgr4/C1s1/Fcgr1/C4b/C1qb/Cfh/C1qc/Ptafr/C1ra/Itgb2/Il10/Icam1/C1rb/Selplg/Krt14/Krt23/C3ar1
mmu05143	African trypanosomiasis	8.69E-05	13	Il6/Hbb-bs/Hba-a2/Hba-a1/Il1b/Hbb-bt/Sele/Vcam1/Tnf/Lama4/Il10/Icam1/Apoa1
mmu05140	Leishmaniasis	9.47E-05	18	Il1b/Ptgs2/Marcksl1/C3/Fcgr3/Il1a/Itgam/Fcgr4/Tnf/Fcgr1/Nos2/Tgfb1/Itgb2/Il10/Tlr2/Cybb/Ncf4/Ncf1
mmu05321	Inflammatory bowel disease	0.000275632	16	Il6/Il1b/Il1a/Tnf/Il18rap/Tgfb1/Il4ra/Foxp3/Il10/Rora/Tlr2/Gata3/Stat4/Maf/Il18r1/Il22
mmu04510	Focal adhesion	0.000302024	34	Thbs1/Col4a1/Tnc/Col4a2/Lamb1/Lamc1/Lama1/Pdgfra/Pgf/Lama4/Flt1/Spp1/Vwf/Itgb8/Fn1/Igf1/Itga5/Col6a2/Col6a4/Pdgfrb/Pdgfc/Col6a1/Kdr/Cav1/Col1a2/Lama5/Pdgfb/Parvb/Col1a1/Rac2/Hgf/Itgb3/Col4a4/Fyn
mmu04657	IL-17 signaling pathway	0.000324392	20	S100a8/S100a9/Cxcl5/Il6/Mmp13/Mmp3/Cxcl2/Csf3/Lcn2/Il1b/Ptgs2/Cxcl1/Mmp9/Cxcl3/Ccl2/Tnf/Ccl11/Cebpb/Fosl1/Mapk15
mmu04630	JAK-STAT signaling pathway	0.000738152	29	Il6/Csf3/Il11/Il19/Osmr/Csf3r/Osm/Pdgfra/Gfap/Il5ra/Csf2rb2/Csf2rb/Socs3/Il6st/Il4ra I/Pdgfrb/fnar2/Il10ra/Il10/Il2ra/Il13ra2/Pdgfb/Stat4/Cntfr/Il7r/Il22/Il2rb/Il20rb/Aox1
mmu04010	MAPK signaling pathway	0.000904161	43	Ereg/Il1b/Il1r1/Dusp1/Il1a/Pdgfra/Pgf/Hspa2/Tnf/Flt1/Hspa1a/Nr4a1/Fgf7/Igf1/Angpt1/Fgfr1/Hspb1/Hspa1b/Efna5/Arrb2/Tgfb1/Stmn1/Tgfbr1/Pdgfrb/Pdgfc/Angpt2/Csf1r/Nras/Cacna2d1/Kdr/Ptpn5/Pdgfb/Dusp2/Fgfr3/Rac2/Map3k8/Hgf/Cacng7/Cacna1e/Map3k6/Pla2g4c/Rps6ka6/Klk1b4
mmu04611	Platelet activation	0.001249729	23	Fga/Fcer1g/Fgg/Nos3/Vwf/Ptgs1/Adcy4/Gucy1b1/Col1a2/Ptgir/F2rl3/F2r/Col3a1/Col1a1/Pik3r6/P2ry12/Itgb3/Lcp2/Pik3r5/Fyn/Fermt3/P2rx1/Pla2g4c
mmu04670	Leukocyte transendothelial migration	0.001551043	22	Mmp9/Cdh5/Vcam1/Itgam/Mmp2/Pecam1/Msn/Esam/Cxcl12/Cldn4/Itgb2/Icam1/Rac2/Cxcr4/Cybb/Cldn5/Jam2/Cldn1/Rassf5/Jam3/Ncf4/Ncf1
mmu04015	Rap1 signaling pathway	0.001960948	33	Thbs1/Fpr1/Itgam/Pdgfra/Pgf/Flt1/Fgf7/Igf1/Angpt1/Fgfr1/Efna5/Pdgfrb/Pdgfc/Angpt2/Adcy4/Itgb2/Csf1r/Nras/Kdr/F2rl3/F2r/Pdgfb/Fgfr3/Rac2/Hgf/Itgb3/Lcp2/Adora2a/Arap3/Cnr1/Rassf5/Pfn2/Klk1b4
mmu04064	NF-kappa B signaling pathway	0.0020404	20	Cxcl2/Il1b/Ptgs2/Cxcl1/Il1r1/Cxcl3/Plau/Tnfsf11/Vcam1/Tnf/Cxcl12/Bcl2a1b/Ccl4/Bcl2a1a/Bcl2a1d/Icam1/Gm10591/Lbp/Gm13304/Ccl21a
mmu04668	TNF signaling pathway	0.0020404	21	Cxcl5/Il6/Mmp3/Cxcl2/Il1b/Ptgs2/Cxcl1/Mmp9/Cxcl3/Ccl2/Sele/Vcam1/Creb3l3/Tnf/Cebpb/Socs3/Mmp14/Icam1/Map3k8/Creb5/Il18r1
mmu05134	Legionellosis	0.002307615	14	Il6/Cxcl2/Il1b/Cxcl1/Cxcl3/C3/Itgam/Hspa2/Tnf/Hspa1a/Hspa1b/Bnip3/Itgb2/Tlr2
mmu04145	Phagosome	0.002320738	29	Thbs1/Olr1/Marco/C3/Fcgr3/Fcgr2b/Msr1/Itgam/Clec7a/Fcgr4/Colec12/Mrc1/Fcgr1/Cd36/Itga5/Mrc2/C1ra/Itgb2/Tubb3/Nos1/C1rb/H2-Q10/Tlr2/Itgb3/Cybb/Pla2r1/Coro1a/Ncf4/Ncf1
mmu05152	Tuberculosis	0.004243105	28	Il6/Il1b/Sphk1/C3/Fcgr3/Clec4e/Fcgr2b/Il1a/Fcer1g/Itgam/Clec7a/Fcgr4/Tnf/Mrc1/Fcgr1/Cebpb/Mrc2/Nos2/Tgfb1/Itgb2/Il10ra/Il10/Card9/Itgax/Tlr2/Lbp/Pla2r1/Coro1a
mmu04926	Relaxin signaling pathway	0.004501477	22	Mmp13/Mmp9/Col4a1/Col4a2/Mmp2/Creb3l3/Nos3/Nos2/Arrb2/Tgfb1/Ednrb/Tgfbr1/Adcy4/Nras/Gng2/Nos1/Col1a2/Col3a1/Col1a1/Col4a4/Creb5/Gng4
mmu05202	Transcriptional misregulation in cancer	0.006992589	32	Il6/Mmp3/Il1r2/Mmp9/Etv4/Plau/Plat/Itgam/Flt1/Fcgr1/Cebpb/Igfbp3/Igf1/Bcl2a1b/Erg/Spi1/Runx1/Bcl2a1a/Bcl2a1d/Csf1r/Nupr1/Rel/Zbtb16/Cd86/Fli1/Zeb1/Prom1/Ccna2/Maf/Runx2/Nr4a3/Il2rb
mmu05205	Proteoglycans in cancer	0.007036418	30	Thbs1/Mmp9/Plaur/Plau/Mmp2/Msn/Dcn/Tnf/Twist2/Timp3/Fzd1/Fn1/Igf1/Itga5/Fgfr1/Tgfb1/Fzd4/Nras/Wnt5a/Kdr/Hcls1/Cav1/Col1a2/Lum/Col1a1/Tlr2/Twist1/Hgf/Itgb3/Wnt11
mmu04350	TGF-beta signaling pathway	0.009765721	17	Inhbb/Thbs1/Grem1/Nbl1/Fst/Dcn/Bmp7/Inhba/Tnf/Ltbp1/Bmpr2/Chrd/Tgfb1/Tgfbr1/Bmp5/Fbn1/Bmp8b
mmu04020	Calcium signaling pathway	0.011240288	33	Sphk1/Bdkrb1/Tacr1/Pdgfra/Ednra/Nos3/Flt1/Fgf7/Fgfr1/Ptafr/Ret/Pde1a/Nos2/Ednrb/Pdgfrb/Pdgfc/Adcy4/Htr4/Kdr/Bdkrb2/Nos1/F2r/Htr7/Pdgfb/Fgfr3/Hgf/Cxcr4/Adora2a/P2rx1/Cacna1e/Avpr1a/Plcd3/Klk1b4
mmu05145	Toxoplasmosis	0.019183988	18	Lamb1/Lamc1/Lama1/Hspa2/Tnf/Lama4/Hspa1a/Nos2/Hspa1b/Tgfb1/Il10ra/Il10/Lama5/Tlr2/Ccr5/Pik3r6/Pik3r5/Igtp
mmu05142	Chagas disease	0.022038178	17	Il6/Serpine1/Il1b/Ccl2/C3/C1qa/Tnf/C1qb/Ccl3/C1qc/Nos2/Tgfb1/Tgfbr1/Il10/Bdkrb2/Ppp2r2b/Tlr2
mmu04072	Phospholipase D signaling pathway	0.025003398	22	Cxcr2/Sphk1/Fcer1g/Pdgfra/Plpp3/Cyth3/Pdgfrb/Pdgfc/Adcy4/Nras/F2r/Pdgfb/Dnm1/Pik3r6/Pik3r5/Grm7/Cyth4/Fyn/Agt/Avpr1a/Pla2g4c/Dnm3
mmu05215	Prostate cancer	0.033625213	16	Mmp3/Il1r2/Mmp9/Plau/Plat/Pdgfra/Creb3l3/Igf1/Fgfr1/Erg/Pdgfrb/Pdgfc/Nras/Pdgfb/Zeb1/Creb5
mmu04620	Toll-like receptor signaling pathway	0.036246562	16	Il6/Il1b/Tnf/Spp1/Ccl3/Ctsk/Ifnar2/Ccl4/Cd80/Cd86/Tlr2/Tlr8/Tlr7/Map3k8/Lbp/Irf5
mmu04672	Intestinal immune network for IgA production	0.037643516	9	Il6/Icos/Cxcl12/Tgfb1/Madcam1/Cd80/Il10/Cd86/Cxcr4

### Mesalazine Triggered Immunosuppressive Response in DSS-Induced Colitis

**FIGURE 2:**
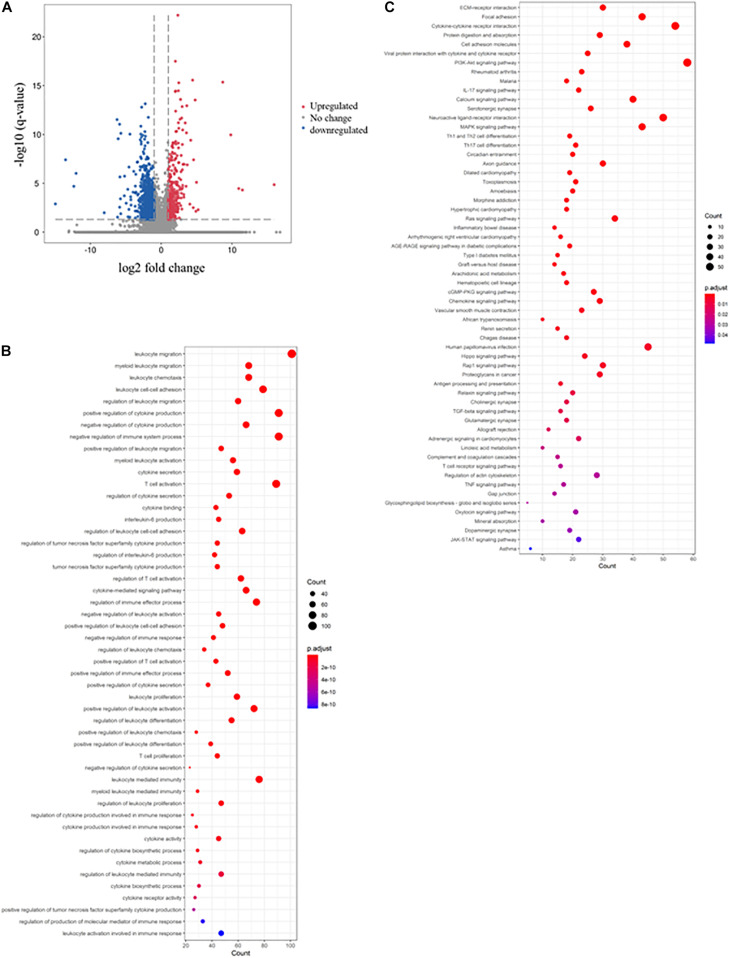
Immunosuppressive effects of mesalazine on DSS-induced colitis. The volcano plot shows the differential expression of genes in the mesalazine-treated colitis mice. The genes with | log_2_ (fold change: mesalazine + DSS/DSS)| > 1 and -log B and H corrected *p*-value > 1.3 were considered DEGs. The red dot represents upregulated genes, the green dot represents downregulated genes, and the gray dot represents genes with no significant change. **(B)** The rich factor plot shows the alteration of biological processes related to immunity in the gut of mesalazine-treated mice with colitis. The size of the dot represents the number of DEGs. The color intensity of the dots represents the significance of the biology processes. **(C)** The rich factor plot showed the alteration of cell signaling pathways related to immunity in the gut of mesalazine-treated colitis mice. The size of the dots represents the number of DEGs. The color intensity of the dots represents the significance of the signaling pathways.

A similar approach was used to determine the immunosuppressive effect of mesalazine on DSS-induced colitis. When we compared the DSS group and DSS + mesalazine group, we identified 1,663 DEGs, of which 262 were upregulated and 1,401 were downregulated (Figure 2A and Supplementary Table 4). In the bioinformatic analysis, we primarily focused on the GO terms and pathways related to immune responses. The results of GO analysis showed that low-dose mesalazine treatment altered different immune processes. This could be due to the regulation of different immune cell types, such as T cells, B cells, natural killer cells, and leukocytes (Figure 2B and Table 3). This results in the regulation of cytokine functions such as cytokine biosynthetic process, cytokine production, cytokine secretion, and cytokine binding (Figure 2B and Table 3), leading to alterations in the immune response of the gut system (Figure 2B and Table 3). The results of KEGG pathway analysis further highlighted the immune-responsive pathways such as ECM-receptor interaction, T-cell receptor signaling pathway, TNF signaling pathway, cytokine-cytokine receptor interaction, chemokine signaling pathway, IL-17 signaling pathway, Th1, Th2, and Th17 cell differentiation, IBD, Hippo signaling pathway, and TGF-beta signaling pathway (Figure 2C and Table 4).

**TABLE 3:** Effects of Mesalazine on the DSS-induced biological inflammatory responses.

GO ID	Description	Adjusted p-value	Number of gene	Gene ID
GO:0050900	leukocyte migration	7.42E-40	101	S100a8/S100a9/Retnlg/Cxcl5/Cxcl2/Cxcr2/Serpine1/Thbs1/Il1b/Fpr2/Cxcl1/Apod/Grem1/Il1r1/Mmp9/Lgmn/Cxcl3/Csf3r/Pla2g7/Ccl2/Trem1/Dusp1/Selp/Bdkrb1/Fcgr3/Sele/Cyp7b1/Tacr1/Il1a/Tnfsf11/Nbl1/Fcer1g/Vcam1/Il33/Itgam/Plvap/Ppbp/Cxcl13/S1pr1/Ednra/Pecam1/Msn/Pgf/Jaml/Tnf/Flt1/Ccr1/Spp1/Ccl11/Ccl3/Pde4b/Ch25h/Pf4/Adam8/Ccl8/Cxcl12/Ptafr/Trpm2/Prex1/Emilin1/Cd300a/Tgfb1/Cxcl16/Ednrb/Spns2/Madcam1/Cmklr1/Ccl4/Mmp14/Itgb2/Wnt5a/Icam1/Sirpa/Ptpn22/Ccr7/Rarres2/Pdgfb/Sell/Ccr2/Trpv4/Bst1/Tlr2/Rac2/Selplg/Adora1/Gata3/P2ry12/Lbp/Itgb3/Gpsm3/Dysf/Podxl/Aif1/Coro1a/Slamf8/Nckap1l/Nkx2-3/Jam3/Cd34/Ccl21a/C3ar1
GO:0097529	myeloid leukocyte migration	1.01E-29	68	S100a8/S100a9/Retnlg/Cxcl5/Cxcl2/Cxcr2/Serpine1/Thbs1/Il1b/Fpr2/Cxcl1/Grem1/Il1r1/Lgmn/Cxcl3/Csf3r/Pla2g7/Ccl2/Trem1/Dusp1/Fcgr3/Il1a/Tnfsf11/Nbl1/Fcer1g/Itgam/Ppbp/Cxcl13/Ednra/Pecam1/Pgf/Jaml/Flt1/Ccr1/Spp1/Ccl11/Ccl3/Pde4b/Pf4/Adam8/Ccl8/Cxcl12/Prex1/Emilin1/Cd300a/Ednrb/Cmklr1/Ccl4/Mmp14/Itgb2/Sirpa/Ccr7/Rarres2/Pdgfb/Sell/Ccr2/Trpv4/Bst1/Rac2/P2ry12/Lbp/Dysf/Aif1/Slamf8/Nckap1l/Jam3/Ccl21a/C3ar1
GO:0030595	leukocyte chemotaxis	3.63E-28	68	S100a8/S100a9/Retnlg/Cxcl5/Cxcl2/Cxcr2/Serpine1/Thbs1/Il1b/Fpr2/Cxcl1/Grem1/Lgmn/Cxcl3/Csf3r/Pla2g7/Ccl2/Trem1/Dusp1/Fcgr3/Cyp7b1/Tnfsf11/Nbl1/Fcer1g/Itgam/Ppbp/Cxcl13/S1pr1/Ednra/Pgf/Jaml/Flt1/Ccr1/Spp1/Ccl11/Ccl3/Pde4b/Ch25h/Pf4/Adam8/Ccl8/Cxcl12/Trpm2/Prex1/Cxcl16/Ednrb/Cmklr1/Ccl4/Itgb2/Wnt5a/Ccr7/Rarres2/Pdgfb/Sell/Ccr2/Trpv4/Bst1/Rac2/Lbp/Gpsm3/Dysf/Aif1/Coro1a/Slamf8/Nckap1l/Jam3/Ccl21a/C3ar1
GO:0007159	leukocyte cell-cell adhesion	4.18E-25	79	S100a8/S100a9/Il6/Olr1/Il1b/Ccl2/Selp/Sele/Tnfsf11/Vcam1/Itgam/Nlrp3/Lrrc32/Pecam1/Msn/Bmp7/Tarm1/Tnf/Ctla4/Arg2/Vnn1/Arg1/Icos/Gpnmb/Cebpb/Adam8/Cxcl12/Igf1/Zc3h12a/Ets1/Itga5/Hlx/Ptafr/Ager/Hsph1/Il6st/Vsir/Cd300a/Tgfb1/Havcr2/Il4ra/Madcam1/Cd4/Runx1/Cd80/Itgb2/Foxp3/Ccdc88b/Icam1/Il2ra/Sirpa/Zbtb16/Cd244a/Ptpn22/Cav1/Ccr7/Myb/Cd86/Sell/Ccr2/Rac2/Selplg/Pik3r6/Gata3/Tigit/Lag3/Il7r/Adora2a/Tnfaip8l2/Fermt3/Aif1/Coro1a/Nr4a3/Cd6/Tnfsf9/Nckap1l/Runx3/Il20rb/Vtcn1
GO:0002685	regulation of leukocyte migration	2.03E-24	60	Cxcr2/Serpine1/Thbs1/Il1b/Fpr2/Apod/Grem1/Il1r1/Mmp9/Lgmn/Pla2g7/Ccl2/Dusp1/Selp/Bdkrb1/Sele/Tacr1/Il1a/Nbl1/Il33/Plvap/Cxcl13/Ednra/Pecam1/Msn/Pgf/Ccr1/Adam8/Cxcl12/Ptafr/Emilin1/Cd300a/Tgfb1/Madcam1/Cmklr1/Ccl4/Mmp14/Wnt5a/Icam1/Ptpn22/Ccr7/Rarres2/Sell/Ccr2/Trpv4/Bst1/Tlr2/Rac2/Adora1/P2ry12/Lbp/Itgb3/Gpsm3/Dysf/Aif1/Slamf8/Nckap1l/Jam3/Ccl21a/C3ar1
GO:0001819	positive regulation of cytokine production	2.74E-23	91	Il1rl1/Il6/Mmp8/Serpine1/Thbs1/Ereg/Il1b/Ptgs2/Il1r1/Sema7a/Mmp12/Ccl2/C3/Fcgr3/Clec4e/Osm/Il1a/Fcer1g/Il33/Clec4n/Nlrp3/Tarm1/Cd200/Hilpda/Tnf/Cebpb/Slc11a1/Ccl3/Pde4b/Pf4/Adam8/Cd36/Sulf1/Gprc5b/Ptafr/Hspb1/Ager/Cd300c2/Egr1/Tgfb1/Havcr2/Il4ra/Arid5a/Ccl4/Runx1/Nfatc4/Ccbe1/Cd84/Csf1r/Foxp3/Ccdc88b/Nras/Wnt5a/Rel/Afap1l2/Il10/Cd244a/Ptpn22/Ccr7/F2r/Cyp1b1/Ccr2/Trpv4/Card9/Lum/Rora/Tlr2/Ccr5/Twist1/Tlr8/Tlr7/Clec5a/Gata3/Tigit/Hgf/Cadm1/Lbp/Cybb/Gpsm3/Spon2/Agt/Wnt11/Il18r1/Nr4a3/Cd6/Tnfsf9/Il20rb/Vtcn1/Cd34/Fgr/C3ar1
GO:0001818	negative regulation of cytokine production	9.33E-23	66	Il1rl1/Il6/Mmp8/Inhbb/Thbs1/Il1r2/Apod/Srgn/Tnfrsf9/Fcgr2b/Hmox1/Il33/Nlrp3/Lrrc32/Tnf/Arg2/Twist2/Arg1/Gpnmb/Slc11a1/Acod1/Klf2/Fn1/Zc3h12a/Angpt1/Fgfr1/Mefv/Ager/Vsir/Arrb2/Tgfb1/Axl/Havcr2/Cmklr1/Prg4/Cd84/Muc16/Foxp3/Rel/Il10/Sirpa/Ptpn22/Clec4a2/Apoa1/Ffar4/Tlr2/Twist1/Tlr8/Ssc5d/Gata3/Tigit/Hgf/Irak3/Lbp/Lag3/Trib2/Sh2d1b1/Wnt11/Syt11/Nav3/Nckap1l/Tnfrsf4/Il20rb/Inpp5d/Fbln1/Cd34
GO:0002683	negative regulation of immune system process	2.77E-21	91	Il1rl1/Cxcr2/Thbs1/Apod/Grem1/Mmp12/Dusp1/Fcgr2b/Hmox1/Nbl1/Fcer1g/Il33/Cd300lf/Sfrp1/Foxf1/Lrrc32/Tmem176a/Tarm1/Cd200/Tnf/Ctla4/Arg2/Twist2/Arg1/Ccr1/Gpnmb/Cebpb/Acod1/Serping1/Pf4/Cxcl12/Igf1/Zc3h12a/Angpt1/C1qc/Hlx/Emilin1/Samsn1/Vsir/Cd300a/Arrb2/Tgfb1/Axl/Alox15/Havcr2/Il4ra/A2m/Runx1/Cd84/Cd80/Foxp3/Tmem176b/Il10/Il2ra/Zbtb16/Il13ra2/Ptpn22/Gpx2/Cd86/Ccr2/Col3a1/Apoa1/Milr1/Adora1/Tigit/Irak3/Lag3/Zfp36l1/Pdcd1/Il7r/Adora2a/Prdm1/Gpr171/Cnr1/Tnfaip8l2/Sh2d1b1/Adgrf5/Gata2/Fbn1/Gal/Slamf8/Pik3ap1/Runx3/Tnfrsf4/Il20rb/Tal1/Klrb1b/Inpp5d/Hist1h4n/Vtcn1/Meis2
GO:0002687	positive regulation of leukocyte migration	1.59E-20	47	Cxcr2/Serpine1/Thbs1/Il1b/Fpr2/Il1r1/Mmp9/Lgmn/Pla2g7/Ccl2/Selp/Bdkrb1/Sele/Tacr1/Il1a/Plvap/Cxcl13/Ednra/Pecam1/Pgf/Ccr1/Adam8/Cxcl12/Ptafr/Tgfb1/Madcam1/Cmklr1/Ccl4/Mmp14/Wnt5a/Icam1/Ccr7/Rarres2/Sell/Ccr2/Trpv4/Tlr2/Rac2/P2ry12/Lbp/Itgb3/Gpsm3/Dysf/Aif1/Nckap1l/Ccl21a/C3ar1
GO:0002274	myeloid leukocyte activation	2.09E-20	56	Cxcl5/Il1rl1/Mmp8/Cxcr2/Thbs1/Fpr2/Fcgr3/Hmox1/Fcer1g/Il33/Itgam/Cd300lf/Foxf1/Cd200/Fcgr4/Clec4d/Pirb/Tyrobp/Slc11a1/Il18rap/Ptafr/Ager/Cd300a/Tgfb1/Havcr2/Il4ra/Spi1/Stx11/Batf/Pla2g3/Cd84/Itgb2/Wnt5a/Il10/Cd244a/Il13ra2/Cd300lb/Ccr2/Rora/Tlr2/Rac2/Tlr8/Tlr7/Milr1/Lbp/Lcp2/Dysf/Cnr1/Adgrf5/Gata2/Aif1/Slc7a2/Nr4a3/Tnfsf9/Fgr/Myo1f
GO:0050663	cytokine secretion	7.30E-20	59	Il1rl1/Il6/Mmp8/Il1b/Chil1/Il1r2/Srgn/Mmp12/Trem1/Abca1/Clec4e/Osm/Tnfrsf9/Il1a/Il33/Clec4n/Nlrp3/Lrrc32/Tnf/Arg2/Ccl3/Fn1/Cd36/Zc3h12a/Angpt1/Nos2/Cd300c2/Havcr2/Il4ra/Arid5a/Cd84/Csf1r/Foxp3/Wnt5a/Il10/Cd244a/Ptpn22/Ccr7/F2r/Trpv4/Apoa1/Ffar4/Tlr2/Ccr5/Twist1/Tlr8/Clec5a/Ssc5d/Gata3/Cadm1/Lcp2/Dysf/Agt/Syt11/Tnfrsf4/Fbln1/Vtcn1/Cd34/Fgr
GO:0042110	T cell activation	1.10E-18	89	Il6/Il1b/Ctla2a/Ccl2/Clec4e/Ctps/Tnfsf11/Fcer1g/Vcam1/Itgam/Nlrp3/Lrrc32/Msn/Tarm1/Jaml/Ctla4/Arg2/Clec4d/Vnn1/Arg1/Icos/Gpnmb/Cebpb/Slc11a1/Adam8/Cxcl12/Igf1/Zc3h12a/Hlx/Ager/Prex1/Hsph1/Il6st/Vsir/Cd300a/Egr1/Tgfb1/Havcr2/Il4ra/Cd4/Stx11/Runx1/Batf/Cd80/Bcl2a1d/Itgb2/Foxp3/Ccdc88b/Icam1/Il2ra/Sirpa/Zbtb16/Cd244a/Ptpn22/Cav1/Ccr7/Satb1/Myb/Clec4a2/Cd86/Ccr2/Rora/Rac2/Zeb1/Pik3r6/Gata3/Tigit/Lag3/Cxcr4/Zfp36l1/Il7r/Adora2a/Prdm1/Tnfaip8l2/Fyn/Fanca/Runx2/Aif1/Treml2/Il18r1/Coro1a/Cd6/Tnfsf9/Nckap1l/Runx3/Nkx2-3/Tnfrsf4/Il20rb/Vtcn1
GO:0050707	regulation of cytokine secretion	1.97E-18	53	Il1rl1/Il6/Mmp8/Il1b/Il1r2/Srgn/Mmp12/Clec4e/Osm/Tnfrsf9/Il1a/Il33/Clec4n/Nlrp3/Lrrc32/Tnf/Arg2/Ccl3/Fn1/Cd36/Zc3h12a/Angpt1/Cd300c2/Havcr2/Il4ra/Arid5a/Cd84/Csf1r/Foxp3/Wnt5a/Il10/Cd244a/Ptpn22/Ccr7/F2r/Trpv4/Apoa1/Ffar4/Tlr2/Ccr5/Twist1/Tlr8/Clec5a/Ssc5d/Gata3/Cadm1/Agt/Syt11/Tnfrsf4/Fbln1/Vtcn1/Cd34/Fgr
GO:0019955	cytokine binding	5.93E-18	43	Il1rl1/Cxcr2/Thbs1/Il1r2/Grem1/Osmr/Il1r1/Csf3r/Tnfrsf9/Tgfbr3/Nbl1/Pdpn/Eng/Lrrc32/Ccr1/Il5ra/Ltbp1/Cd36/Bmpr2/Ackr3/Ager/Il6st/Tgfbr1/Cd4/Ifnar2/Fzd4/A2m/Csf1r/Il10ra/Ltbp3/Il2ra/Il13ra2/Ccr7/Ccr2/Ccr5/Itgb3/Cxcr4/Cntfr/Sostdc1/Il18r1/Il2rb/Il20rb/Nrros
GO:0032635	interleukin-6 production	5.70E-18	45	Il6/Mmp8/Ereg/Il1b/Il19/Il1a/Fcer1g/Il33/Tnf/Cebpb/Klf2/Il18rap/Cd36/Zc3h12a/Ptafr/Nos2/Arrb2/Havcr2/Arid5a/Prg4/Cd84/Muc16/Foxp3/Wnt5a/Afap1l2/Il10/Cd300ld/Sirpa/Ptpn22/F2r/Trpv4/Card9/Tlr2/Ccr5/Twist1/Tlr8/Tlr7/Hgf/Irak3/Lbp/Spon2/Syt11/Tnfsf9/Nckap1l/Inpp5d
GO:1903037	regulation of leukocyte cell-cell adhesion	1.07E-17	63	Il6/Il1b/Ccl2/Tnfsf11/Vcam1/Nlrp3/Lrrc32/Tarm1/Tnf/Ctla4/Arg2/Vnn1/Arg1/Icos/Gpnmb/Cebpb/Adam8/Cxcl12/Igf1/Zc3h12a/Ets1/Hlx/Ptafr/Ager/Hsph1/Il6st/Vsir/Cd300a/Tgfb1/Havcr2/Il4ra/Cd4/Runx1/Cd80/Foxp3/Ccdc88b/Icam1/Il2ra/Sirpa/Zbtb16/Cd244a/Ptpn22/Cav1/Ccr7/Myb/Cd86/Ccr2/Pik3r6/Gata3/Tigit/Lag3/Il7r/Adora2a/Tnfaip8l2/Aif1/Coro1a/Nr4a3/Cd6/Tnfsf9/Nckap1l/Runx3/Il20rb/Vtcn1
GO:1903555	regulation of tumor necrosis factor superfamily cytokine production	7.43E-17	44	Mmp8/Thbs1/Ccl2/Fcgr3/Fcer1g/Ltf/Arg2/Twist2/Gpnmb/Ccl3/Pf4/Adam8/Cd36/Zc3h12a/Angpt1/Ptafr/Hspb1/Vsir/Arrb2/Axl/Havcr2/Arid5a/Ccl4/Nfatc4/Cd84/Foxp3/Wnt5a/Il10/Cd300ld/Sirpa/Ptpn22/Ccr7/Clec4a2/Ccr2/Card9/Tlr2/Ccr5/Twist1/Irak3/Lbp/Cybb/Spon2/Syt11/Cd34
GO:0032675	regulation of interleukin-6 production	1.66E-16	42	Il6/Mmp8/Ereg/Il1b/Il1a/Fcer1g/Il33/Tnf/Cebpb/Klf2/Cd36/Zc3h12a/Ptafr/Arrb2/Havcr2/Arid5a/Prg4/Cd84/Muc16/Foxp3/Wnt5a/Afap1l2/Il10/Cd300ld/Sirpa/Ptpn22/F2r/Trpv4/Card9/Tlr2/Ccr5/Twist1/Tlr8/Tlr7/Hgf/Irak3/Lbp/Spon2/Syt11/Tnfsf9/Nckap1l/Inpp5d
GO:0071706	tumor necrosis factor superfamily cytokine production	1.66E-16	44	Mmp8/Thbs1/Ccl2/Fcgr3/Fcer1g/Ltf/Arg2/Twist2/Gpnmb/Ccl3/Pf4/Adam8/Cd36/Zc3h12a/Angpt1/Ptafr/Hspb1/Vsir/Arrb2/Axl/Havcr2/Arid5a/Ccl4/Nfatc4/Cd84/Foxp3/Wnt5a/Il10/Cd300ld/Sirpa/Ptpn22/Ccr7/Clec4a2/Ccr2/Card9/Tlr2/Ccr5/Twist1/Irak3/Lbp/Cybb/Spon2/Syt11/Cd34
GO:0050863	regulation of T cell activation	1.09E-15	62	Il6/Il1b/Ctla2a/Ccl2/Tnfsf11/Vcam1/Nlrp3/Lrrc32/Tarm1/Ctla4/Arg2/Vnn1/Arg1/Icos/Gpnmb/Cebpb/Adam8/Igf1/Zc3h12a/Hlx/Ager/Hsph1/Il6st/Vsir/Cd300a/Tgfb1/Havcr2/Il4ra/Cd4/Runx1/Cd80/Foxp3/Ccdc88b/Il2ra/Sirpa/Zbtb16/Cd244a/Ptpn22/Cav1/Ccr7/Myb/Cd86/Ccr2/Rac2/Zeb1/Pik3r6/Gata3/Tigit/Lag3/Il7r/Adora2a/Prdm1/Tnfaip8l2/Fanca/Aif1/Coro1a/Cd6/Tnfsf9/Nckap1l/Runx3/Il20rb/Vtcn1
GO:0019221	cytokine-mediated signaling pathway	1.19E-15	66	Cxcl5/Il6/Cxcl2/Cxcr2/Ifitm1/Ereg/Il1b/Cxcl1/Il1r2/Osmr/Il1r1/Mmp12/Cxcl3/Ifitm6/Sphk1/Ccl2/Osm/Il1a/Tnfsf11/Fcer1g/Plvap/Slit3/Cd300lf/Ppbp/Cxcl13/Tnf/Arg1/Pirb/Ccr1/Trem2/Ccl11/Ccl3/Csf2rb2/Csf2rb/Pf4/Ccl8/Il18rap/Cxcl12/Angpt1/Ackr3/Il6st/Egr1/Axl/Cd4/Ifnar2/Ccl4/Csf1r/Wnt5a/Il10ra/Cav1/Numbl/Pdgfb/Ccr2/Apoa1/Irak3/Stat4/Sh2b2/Cxcr4/Cntfr/F3/Ptprn/Il18r1/Irf5/Il2rb/Il20rb/Ccl21a
GO:0002697	regulation of immune effector process	2.58E-15	74	Cxcl5/Il6/Il1b/Cxcl1/Il1r1/Sema7a/Mmp12/Ccl2/C3/Fcgr3/Fcgr2b/Hmox1/Fcer1g/Il33/Itgam/Nlrp3/Foxf1/Tnf/Arg1/Fcgr1/Serping1/Il18rap/Cd36/Zc3h12a/Gprc5b/Angpt1/Cfh/Hlx/Ptafr/Ager/Vsir/Cd300a/Arrb2/Tgfb1/Havcr2/Il4ra/A2m/Arid5a/Cd84/Itgb2/Foxp3/Wnt5a/Il10/Il2ra/Cd55/Cd244a/Il13ra2/Ptpn22/Ccr7/Myb/H2-Q10/Ccr2/Apoa1/Tlr2/Rac2/Pik3r6/Gata3/Irak3/Cadm1/Lbp/Lag3/Il7r/Sh2d1b1/Spon2/Gata2/Clcf1/Il18r1/Nr4a3/Slamf8/C6/Tnfrsf4/Il20rb/Klrb1b/Fgr
GO:0002695	negative regulation of leukocyte activation	4.08E-15	45	Fcgr2b/Hmox1/Cd300lf/Sfrp1/Foxf1/Lrrc32/Tarm1/Cd200/Ctla4/Arg2/Arg1/Gpnmb/Cebpb/Zc3h12a/Hlx/Samsn1/Vsir/Cd300a/Tgfb1/Axl/Havcr2/Il4ra/Runx1/Cd84/Cd80/Foxp3/Il10/Il2ra/Il13ra2/Ptpn22/Cd86/Ccr2/Milr1/Tigit/Lag3/Adora2a/Prdm1/Cnr1/Tnfaip8l2/Adgrf5/Gal/Runx3/Il20rb/Inpp5d/Vtcn1
GO:1903039	positive regulation of leukocyte cell-cell adhesion	4.31E-15	48	Il6/Il1b/Ccl2/Tnfsf11/Vcam1/Nlrp3/Tnf/Vnn1/Icos/Adam8/Igf1/Ets1/Hlx/Ptafr/Ager/Hsph1/Il6st/Vsir/Tgfb1/Havcr2/Il4ra/Cd4/Runx1/Cd80/Foxp3/Ccdc88b/Icam1/Il2ra/Sirpa/Zbtb16/Cd244a/Ptpn22/Cav1/Ccr7/Myb/Cd86/Ccr2/Pik3r6/Gata3/Il7r/Aif1/Coro1a/Nr4a3/Cd6/Tnfsf9/Nckap1l/Runx3/Vtcn1
GO:0050777	negative regulation of immune response	6.11E-15	41	Il1rl1/Mmp12/Fcgr2b/Hmox1/Il33/Foxf1/Tnf/Ctla4/Arg2/Arg1/Ccr1/Acod1/Serping1/Zc3h12a/Angpt1/Hlx/Samsn1/Vsir/Cd300a/Arrb2/Tgfb1/Alox15/Havcr2/Il4ra/A2m/Cd84/Foxp3/Il10/Il2ra/Il13ra2/Gpx2/Ccr2/Col3a1/Apoa1/Irak3/Il7r/Sh2d1b1/Slamf8/Il20rb/Klrb1b/Inpp5d
GO:0002688	regulation of leukocyte chemotaxis	8.45E-14	34	Cxcr2/Serpine1/Thbs1/Il1b/Fpr2/Grem1/Lgmn/Pla2g7/Ccl2/Dusp1/Nbl1/Cxcl13/Ednra/Pgf/Ccr1/Cxcl12/Cmklr1/Ccl4/Wnt5a/Ccr7/Rarres2/Sell/Ccr2/Trpv4/Bst1/Rac2/Lbp/Gpsm3/Dysf/Aif1/Slamf8/Nckap1l/Jam3/C3ar1
GO:0050870	positive regulation of T cell activation	7.75E-13	43	Il6/Il1b/Ccl2/Tnfsf11/Vcam1/Nlrp3/Vnn1/Icos/Adam8/Igf1/Hlx/Ager/Hsph1/Il6st/Vsir/Tgfb1/Havcr2/Il4ra/Cd4/Runx1/Cd80/Foxp3/Ccdc88b/Il2ra/Sirpa/Zbtb16/Cd244a/Ptpn22/Cav1/Ccr7/Myb/Cd86/Ccr2/Pik3r6/Gata3/Il7r/Aif1/Coro1a/Cd6/Tnfsf9/Nckap1l/Runx3/Vtcn1
GO:0002699	positive regulation of immune effector process	8.87E-13	52	Il6/Il1b/Cxcl1/Il1r1/Sema7a/Ccl2/C3/Fcgr3/Hmox1/Fcer1g/Il33/Itgam/Nlrp3/Foxf1/Tnf/Arg1/Fcgr1/Il18rap/Cd36/Gprc5b/Hlx/Ptafr/Cd300a/Tgfb1/Il4ra/Arid5a/Cd84/Itgb2/Foxp3/Wnt5a/Cd244a/Il13ra2/Ptpn22/Ccr7/Myb/H2-Q10/Ccr2/Tlr2/Rac2/Gata3/Cadm1/Lbp/Lag3/Sh2d1b1/Spon2/Gata2/Clcf1/Il18r1/Nr4a3/C6/Tnfrsf4/Fgr
GO:0050715	positive regulation of cytokine secretion	1.17E-12	37	Il1rl1/Mmp8/Il1b/Mmp12/Clec4e/Osm/Il1a/Il33/Clec4n/Nlrp3/Tnf/Ccl3/Cd36/Cd300c2/Havcr2/Il4ra/Arid5a/Cd84/Csf1r/Wnt5a/Il10/Cd244a/Ptpn22/Ccr7/F2r/Trpv4/Tlr2/Ccr5/Twist1/Tlr8/Clec5a/Gata3/Cadm1/Agt/Vtcn1/Cd34/Fgr
GO:0070661	leukocyte proliferation	1.27E-12	59	Il6/Il1b/Grem1/Tacr1/Fcgr2b/Ctps/Tnfsf11/Vcam1/Il33/Itgam/Lrrc32/Msn/Tarm1/Ctla4/Arg2/Arg1/Gpnmb/Cebpb/Slc11a1/Cxcl12/Igf1/Ager/Il6st/Vsir/Cd300a/Tgfb1/Havcr2/Cd4/Tac1/Cd80/Itgb2/Foxp3/Ccdc88b/Il10/Il2ra/Cd244a/Ptpn22/Ccr7/Satb1/Cd86/Ccr2/Bst1/Rac2/Cxcr4/Il7r/Prdm1/Fyn/Ocstamp/Aif1/Clcf1/Gal/Coro1a/Cd6/Tnfsf9/Nckap1l/Tnfrsf4/Il20rb/Inpp5d/Vtcn1
GO:0002696	positive regulation of leukocyte activation	1.74E-12	72	Il1rl1/Il6/Mmp8/Thbs1/Il1b/Ccl2/Tacr1/Tnfsf11/Fcer1g/Vcam1/Il33/Itgam/Nlrp3/Clec4d/Vnn1/Icos/Adam8/Igf1/Hlx/Ptafr/Ager/Hsph1/Il6st/Vsir/Ighv1-72/Tgfb1/Axl/Havcr2/Il4ra/Cd4/Runx1/Mmp14/Tac1/Cd80/Itgb2/Foxp3/Ccdc88b/Wnt5a/Il10/Il2ra/Sirpa/Zbtb16/Cd244a/Cd300lb/Ptpn22/Cav1/Ccr7/Myb/Ighg2c/Cd86/Ccr2/Bst1/Trdc/Pik3r6/Gata3/Lbp/Il7r/Prdm1/Gata2/Aif1/Clcf1/Coro1a/Nr4a3/Cd6/Tnfsf9/Ighv1-55/Nckap1l/Runx3/Tnfrsf4/Inpp5d/Vtcn1/Fgr
GO:1902105	regulation of leukocyte differentiation	1.94E-12	55	Il6/Il1b/Ctla2a/Tnfsf11/Sfrp1/Nlrp3/Rbp1/Tmem176a/Tnf/Ctla4/Vnn1/Ccr1/Tyrobp/Cebpb/Ccl3/Pf4/Adam8/Zc3h12a/C1qc/Hlx/Ager/Vsir/Tgfb1/Axl/Il4ra/Cd4/Il34/Runx1/Mmp14/Csf1r/Foxp3/Tmem176b/Il2ra/Zbtb16/Hcls1/Ccr7/Myb/Ccr2/Fgfr3/Zeb1/Pik3r6/Gata3/Itgb3/Zfp36l1/Il7r/Prdm1/Fanca/Gata2/Ocstamp/Fbn1/Tnfsf9/Nckap1l/Runx3/Tal1/Inpp5d
GO:0002690	positive regulation of leukocyte chemotaxis	2.80E-12	28	Cxcr2/Serpine1/Thbs1/Il1b/Fpr2/Lgmn/Pla2g7/Ccl2/Cxcl13/Ednra/Pgf/Ccr1/Cxcl12/Cmklr1/Ccl4/Wnt5a/Ccr7/Rarres2/Sell/Ccr2/Trpv4/Rac2/Lbp/Gpsm3/Dysf/Aif1/Nckap1l/C3ar1
GO:1902107	positive regulation of leukocyte differentiation	5.66E-12	39	Il6/Il1b/Tnfsf11/Nlrp3/Tnf/Vnn1/Ccr1/Ccl3/Pf4/Adam8/Hlx/Ager/Vsir/Tgfb1/Axl/Il4ra/Cd4/Il34/Runx1/Mmp14/Csf1r/Foxp3/Il2ra/Zbtb16/Hcls1/Ccr7/Myb/Ccr2/Pik3r6/Gata3/Itgb3/Zfp36l1/Il7r/Prdm1/Ocstamp/Tnfsf9/Nckap1l/Runx3/Inpp5d
GO:0042098	T cell proliferation	5.80E-12	44	Il6/Il1b/Ctps/Vcam1/Itgam/Lrrc32/Msn/Tarm1/Ctla4/Arg2/Arg1/Gpnmb/Cebpb/Slc11a1/Cxcl12/Igf1/Ager/Il6st/Vsir/Tgfb1/Havcr2/Cd4/Cd80/Itgb2/Foxp3/Ccdc88b/Il2ra/Cd244a/Ptpn22/Ccr7/Satb1/Cd86/Ccr2/Rac2/Cxcr4/Fyn/Aif1/Coro1a/Cd6/Tnfsf9/Nckap1l/Tnfrsf4/Il20rb/Vtcn1
GO:0050710	negative regulation of cytokine secretion	6.64E-12	23	Il6/Il1r2/Srgn/Tnfrsf9/Nlrp3/Lrrc32/Tnf/Arg2/Fn1/Zc3h12a/Angpt1/Cd84/Foxp3/Il10/Ptpn22/Apoa1/Ffar4/Tlr8/Ssc5d/Syt11/Tnfrsf4/Fbln1/Cd34
GO:0002443	leukocyte mediated immunity	9.26E-12	76	Cxcl5/Il6/Il1b/Cxcl1/Il1r1/Trem1/C3/Fcgr3/Fcgr2b/Hmox1/C1qa/Fcer1g/Itgam/Nlrp3/Foxf1/Tnf/C1s1/Arg1/Pirb/Fcgr1/C4b/C1qb/Slc11a1/Serping1/Il18rap/C1qc/Ptafr/Ager/Vsir/Ighv1-72/Cd300a/Arrb2/Tgfb1/Havcr2/Il4ra/C1ra/Arid5a/Stx11/Batf/Pla2g3/Cd84/Itgb2/Foxp3/Icam1/Rftn1/Cd55/Il13ra2/Cd300lb/Ighg2c/C1rb/H2-Q10/Ccr2/Tlr2/Rac2/Trdc/Milr1/Pik3r6/Gata3/Cadm1/Lag3/Il7r/Sh2d1b1/Spon2/Gata2/Clcf1/Il18r1/Coro1a/Nr4a3/Ighv1-55/Il20rb/Klrb1b/Inpp5d/Exo1/Ncf1/Fgr/Myo1f
GO:0002444	myeloid leukocyte mediated immunity	1.05E-11	29	Cxcl5/Cxcl1/Trem1/C3/Fcgr3/Hmox1/Fcer1g/Itgam/Foxf1/Arg1/Fcgr1/Ptafr/Cd300a/Il4ra/Stx11/Pla2g3/Cd84/Itgb2/Il13ra2/Cd300lb/Ccr2/Rac2/Milr1/Spon2/Gata2/Nr4a3/Ncf1/Fgr/Myo1f
GO:0070663	regulation of leukocyte proliferation	1.75E-11	47	Il6/Il1b/Grem1/Tacr1/Fcgr2b/Vcam1/Lrrc32/Tarm1/Ctla4/Arg2/Arg1/Gpnmb/Cebpb/Igf1/Ager/Il6st/Vsir/Cd300a/Tgfb1/Havcr2/Cd4/Tac1/Cd80/Foxp3/Ccdc88b/Il10/Il2ra/Cd244a/Ptpn22/Ccr7/Cd86/Ccr2/Bst1/Rac2/Prdm1/Ocstamp/Aif1/Clcf1/Gal/Coro1a/Cd6/Tnfsf9/Nckap1l/Tnfrsf4/Il20rb/Inpp5d/Vtcn1
GO:0002718	regulation of cytokine production involved in immune response	3.32E-11	25	Il6/Il1b/Il1r1/Sema7a/Hmox1/Fcer1g/Nlrp3/Tnf/Arg1/Cd36/Gprc5b/Angpt1/Vsir/Tgfb1/Arid5a/Foxp3/Wnt5a/Il10/Apoa1/Tlr2/Gata3/Irak3/Spon2/Il18r1/Nr4a3
GO:0002367	cytokine production involved in immune response	3.57E-11	28	Il6/Il1b/Il1r1/Sema7a/Trem1/Hmox1/Fcer1g/Nlrp3/Tnf/Arg1/Slc11a1/Il18rap/Cd36/Gprc5b/Angpt1/Vsir/Tgfb1/Arid5a/Foxp3/Wnt5a/Il10/Apoa1/Tlr2/Gata3/Irak3/Spon2/Il18r1/Nr4a3
GO:0005125	cytokine activity	5.13E-11	45	Cxcl5/Il6/Cxcl2/Csf3/Inhbb/Timp1/Il11/Il1b/Il19/Cxcl1/Grem1/Cxcl3/Ccl2/Osm/Il1a/Tnfsf11/Il33/Ppbp/Cxcl13/Bmp3/Bmp7/Inhba/Tnf/Spp1/Ccl11/Ccl3/Pf4/Ccl8/Cxcl12/Tgfb1/Cxcl16/Cmtm3/Il34/Ccl4/Bmp1/Wnt5a/Il10/Bmp5/Scgb3a1/Sectm1a/Clcf1/Bmp8b/Il22/Tnfsf9/Ccl21a
GO:0042035	regulation of cytokine biosynthetic process	3.97E-11	29	Il6/Inhbb/Thbs1/Ereg/Il1b/Fcgr3/Il1a/Tnf/Cebpb/Ptafr/Hspb1/Egr1/Prg4/Muc16/Foxp3/Wnt5a/Rel/Il10/Ccr2/Card9/Tlr2/Tlr8/Tlr7/Gata3/Lbp/Lag3/Trib2/Cybb/Inpp5d
GO:0042107	cytokine metabolic process	6.78E-11	31	Il6/Inhbb/Thbs1/Ereg/Il1b/Il19/Trem1/Fcgr3/Il1a/Tnf/Cebpb/Ptafr/Hspb1/Egr1/Prg4/Muc16/Foxp3/Wnt5a/Rel/Il10/Ccr2/Card9/Tlr2/Tlr8/Tlr7/Gata3/Lbp/Lag3/Trib2/Cybb/Inpp5d
GO:0002703	regulation of leukocyte mediated immunity	1.10E-10	47	Cxcl5/Il6/Il1b/Cxcl1/Il1r1/C3/Fcgr3/Fcgr2b/Hmox1/Fcer1g/Itgam/Nlrp3/Foxf1/Tnf/Arg1/Fcgr1/Il18rap/Ptafr/Ager/Vsir/Cd300a/Arrb2/Tgfb1/Havcr2/Il4ra/Arid5a/Cd84/Itgb2/Foxp3/Cd55/Il13ra2/H2- Q10/Ccr2/Tlr2/Rac2/Pik3r6/Gata3/Cadm1/Lag3/Il7r/Sh2d1b1/Gata2/Clcf1/Il18r1/Il20rb/Klrb1b/Fgr
GO:0042089	cytokine biosynthetic process	1.32E-10	30	Il6/Inhbb/Thbs1/Ereg/Il1b/Il19/Fcgr3/Il1a/Tnf/Cebpb/Ptafr/Hspb1/Egr1/Prg4/Muc16/Foxp3/Wnt5a/Rel/Il10/Ccr2/Card9/Tlr2/Tlr8/Tlr7/Gata3/Lbp/Lag3/Trib2/Cybb/Inpp5d
GO:0004896	cytokine receptor activity	2.43E-10	27	Il1rl1/Cxcr2/Il1r2/Osmr/Il1r1/Csf3r/Ccr1/Il5ra/Csf2rb2/Csf2rb/Il18rap/Ackr3/Il6st/Il4ra/Cd4/Ifnar2/Il10ra/Il2ra/Il13ra2/Ccr7/Ccr2/Ccr5/Cxcr4/Cntfr/Il7r/Il18r1/Il2rb
GO:1903557	positive regulation of tumor necrosis factor superfamily cytokine production	2.90E-10	26	Mmp8/Thbs1/Ccl2/Fcgr3/Fcer1g/Ccl3/Pf4/Adam8/Cd36/Ptafr/Hspb1/Havcr2/Arid5a/Ccl4/Nfatc4/Cd84/Wnt5a/Ccr7/Ccr2/Card9/Tlr2/Ccr5/Twist1/Lbp/Cybb/Spon2
GO:0002700	regulation of production of molecular mediator of immune response	4.64E-10	33	Il6/Il1b/Il1r1/Sema7a/Fcgr2b/Hmox1/Fcer1g/Il33/Nlrp3/Tnf/Arg1/Cd36/Gprc5b/Angpt1/Vsir/Tgfb1/Il4ra/Arid5a/Foxp3/Wnt5a/Il10/Cd244a/Il13ra2/Ptpn22/Apoa1/Tlr2/Gata3/Irak3/Spon2/Clcf1/Il18r1/Nr4a3/Tnfrsf4
GO:0002366	leukocyte activation involved in immune response	4.79E-10	47	Il6/Clec4e/Hmox1/Fcer1g/Il33/Itgam/Nlrp3/Foxf1/Clec4d/Tyrobp/Slc11a1/Zc3h12a/Hlx/Ptafr/Cd300a/Tgfb1/Havcr2/Il4ra/Stx11/Batf/Pla2g3/Cd84/Itgb2/Foxp3/Icam1/Itm2a/Cd244a/Il13ra2/Ccr7/Myb/Ccr2/Rora/Rac2/Milr1/Gata3/Lbp/Dysf/Sh2d1b1/Gata2/Clcf1/Il18r1/Coro1a/Nr4a3/Nkx2-3/Exo1/Fgr/Myo1f
GO:0002263	cell activation involved in immune response	8.10E-10	47	Il6/Clec4e/Hmox1/Fcer1g/Il33/Itgam/Nlrp3/Foxf1/Clec4d/Tyrobp/Slc11a1/Zc3h12a/Hlx/Ptafr/Cd300a/Tgfb1/Havcr2/Il4ra/Stx11/Batf/Pla2g3/Cd84/Itgb2/Foxp3/Icam1/Itm2a/Cd244a/Il13ra2/Ccr7/Myb/Ccr2/Rora/Rac2/Milr1/Gata3/Lbp/Dysf/Sh2d1b1/Gata2/Clcf1/Il18r1/Coro1a/Nr4a3/Nkx2-3/Exo1/Fgr/Myo1f
GO:0002275	myeloid cell activation involved in immune response	1.15E-09	24	Hmox1/Fcer1g/Il33/Itgam/Foxf1/Tyrobp/Ptafr/Cd300a/Havcr2/Il4ra/Stx11/Pla2g3/Cd84/Itgb2/Il13ra2/Ccr2/Rac2/Milr1/Lbp/Dysf/Gata2/Nr4a3/Fgr/Myo1f
GO:0002698	negative regulation of immune effector process	2.05E-09	30	Fcgr2b/Hmox1/Il33/Foxf1/Tnf/Arg1/Serping1/Zc3h12a/Angpt1/Hlx/Vsir/Cd300a/Arrb2/Tgfb1/Havcr2/Il4ra/A2m/Cd84/Foxp3/Il10/Il2ra/Il13ra2/Ccr2/Apoa1/Irak3/Il7r/Sh2d1b1/Slamf8/Il20rb/Klrb1b
GO:0002573	myeloid leukocyte differentiation	2.65E-09	40	Csf3/Tnfsf11/Fcer1g/Itgam/Cd300lf/Sfrp1/Rbp1/Tnf/Pirb/Ccr1/Trem2/Tyrobp/Cebpb/Ccl3/Pf4/Adam8/C1qc/Tgfb1/Spi1/Cd4/Il34/Runx1/Batf/Csf1r/Hcls1/Ccr7/Fam20c/Fgfr3/Tlr2/Gata3/Itgb3/Zfp36l1/Gata2/Ocstamp/Fbn1/Tnfsf9/Nkx2-3/Tal1/Inpp5d/Nrros
GO:0046631	alpha-beta T cell activation	3.93E-09	33	Il6/Nlrp3/Tarm1/Arg2/Cebpb/Zc3h12a/Hlx/Ager/Hsph1/Vsir/Cd300a/Tgfb1/Il4ra/Runx1/Batf/Cd80/Foxp3/Zbtb16/Cd244a/Ptpn22/Ccr7/Satb1/Myb/Clec4a2/Ccr2/Rora/Gata3/Adora2a/Prdm1/Il18r1/Nckap1l/Runx3/Nkx2-3
GO:0046634	regulation of alpha-beta T cell activation	6.42E-09	26	Il6/Nlrp3/Tarm1/Arg2/Zc3h12a/Hlx/Ager/Hsph1/Vsir/Cd300a/Tgfb1/Il4ra/Runx1/Cd80/Foxp3/Zbtb16/Cd244a/Ptpn22/Ccr7/Myb/Ccr2/Gata3/Adora2a/Prdm1/Nckap1l/Runx3
GO:0002886	regulation of myeloid leukocyte mediated immunity	9.27E-09	20	Cxcl5/Cxcl1/C3/Fcgr3/Hmox1/Fcer1g/Itgam/Foxf1/Arg1/Fcgr1/Ptafr/Cd300a/Il4ra/Cd84/Itgb2/Il13ra2/Ccr2/Rac2/Gata2/Fgr
GO:0042129	regulation of T cell proliferation	1.15E-08	34	Il6/Il1b/Vcam1/Lrrc32/Tarm1/Ctla4/Arg2/Arg1/Gpnmb/Cebpb/Igf1/Ager/Il6st/Vsir/Tgfb1/Havcr2/Cd4/Cd80/Foxp3/Ccdc88b/Il2ra/Cd244a/Ptpn22/Ccr7/Cd86/Ccr2/Rac2/Aif1/Coro1a/Cd6/Tnfsf9/Nckap1l/Il20rb/Vtcn1
GO:1903038	negative regulation of leukocyte cell-cell adhesion	1.24E-08	29	Lrrc32/Tarm1/Ctla4/Arg2/Arg1/Gpnmb/Cebpb/Adam8/Cxcl12/Zc3h12a/Hlx/Vsir/Cd300a/Tgfb1/Havcr2/Il4ra/Runx1/Cd80/Foxp3/Il2ra/Ptpn22/Cd86/Tigit/Lag3/Adora2a/Tnfaip8l2/Runx3/Il20rb/Vtcn1
GO:0032715	negative regulation of interleukin-6 production	1.65E-08	17	Tnf/Klf2/Zc3h12a/Arrb2/Havcr2/Prg4/Cd84/Muc16/Foxp3/Il10/Sirpa/Ptpn22/Hgf/Irak3/Syt11/Nckap1l/Inpp5d
GO:0050868	negative regulation of T cell activation	2.30E-08	27	Lrrc32/Tarm1/Ctla4/Arg2/Arg1/Gpnmb/Cebpb/Zc3h12a/Hlx/Vsir/Cd300a/Tgfb1/Havcr2/Il4ra/Runx1/Cd80/Foxp3/Il2ra/Ptpn22/Cd86/Tigit/Lag3/Adora2a/Tnfaip8l2/Runx3/Il20rb/Vtcn1
GO:0070664	negative regulation of leukocyte proliferation	2.51E-08	23	Grem1/Fcgr2b/Lrrc32/Tarm1/Ctla4/Arg2/Arg1/Gpnmb/Cebpb/Vsir/Cd300a/Tgfb1/Havcr2/Cd80/Foxp3/Il10/Il2ra/Cd86/Prdm1/Gal/Il20rb/Inpp5d/Vtcn1
GO:0043299	leukocyte degranulation	2.73E-08	20	Hmox1/Fcer1g/Itgam/Foxf1/Ptafr/Cd300a/Il4ra/Stx11/Pla2g3/Cd84/Itgb2/Il13ra2/Ccr2/Rac2/Milr1/Gata2/Coro1a/Nr4a3/Fgr/Myo1f
GO:0071347	cellular response to interleukin-1	2.97E-08	21	Il6/Saa3/Serpine1/Il1r2/Il1r1/Ccl2/Il1a/Ccl11/Cebpb/Ccl3/Acod1/Ccl8/Adamts7/Fn1/Zc3h12a/Egr1/Ccl4/Rora/Irak3/Adamts12/Ccl21a
GO:0032611	interleukin-1 beta production	3.50E-08	20	Il1b/Sphk1/Abca1/Nlrp3/Arg2/Ccl3/Cd36/Zc3h12a/Mefv/Hspb1/Egr1/Arrb2/Wnt5a/Ccr7/F2r/Apoa1/Tlr2/Ccr5/Tlr8/S1pr3
GO:0035710	CD4-positive, alpha-beta T cell activation	3.65E-08	24	Il6/Nlrp3/Tarm1/Arg2/Cebpb/Zc3h12a/Hlx/Ager/Vsir/Tgfb1/Il4ra/Runx1/Batf/Foxp3/Ccr7/Satb1/Myb/Ccr2/Rora/Gata3/Il18r1/Nckap1l/Runx3/Nkx2-3
GO:0032755	positive regulation of interleukin-6 production	4.77E-08	23	Il6/Mmp8/Ereg/Il1b/Il1a/Fcer1g/Il33/Tnf/Cd36/Ptafr/Arid5a/Wnt5a/F2r/Trpv4/Card9/Tlr2/Ccr5/Twist1/Tlr8/Tlr7/Lbp/Spon2/Tnfsf9
GO:1903556	negative regulation of tumor necrosis factor superfamily cytokine production	5.69E-08	18	Arg2/Twist2/Gpnmb/Zc3h12a/Vsir/Arrb2/Axl/Havcr2/Foxp3/Il10/Sirpa/Ptpn22/Clec4a2/Twist1/Irak3/Lbp/Syt11/Cd34
GO:0030217	T cell differentiation	7.12E-08	43	Il6/Il1b/Ctla2a/Clec4e/Fcer1g/Nlrp3/Ctla4/Clec4d/Vnn1/Adam8/Zc3h12a/Hlx/Prex1/Vsir/Egr1/Tgfb1/Il4ra/Cd4/Runx1/Batf/Bcl2a1d/Foxp3/Il2ra/Zbtb16/Ptpn22/Ccr7/Satb1/Myb/Ccr2/Rora/Zeb1/Pik3r6/Gata3/Zfp36l1/Il7r/Prdm1/Fanca/Runx2/Il18r1/Tnfsf9/Nckap1l/Runx3/Nkx2-3
GO:0032612	interleukin-1 production	9.20E-08	22	Il1b/Il1r2/Sphk1/Abca1/Nlrp3/Arg2/Ccl3/Cd36/Zc3h12a/Mefv/Hspb1/Egr1/Arrb2/Havcr2/Wnt5a/Ccr7/F2r/Apoa1/Tlr2/Ccr5/Tlr8/S1pr3
GO:0005126	cytokine receptor binding	1.39E-07	50	Cxcl5/Il6/Cxcl2/Csf3/Inhbb/Il11/Il1b/Cxcl1/Grem1/Cxcl3/Ccl2/Lrg1/Osm/Il1a/Tgfbr3/Tnfsf11/Eng/Cd300lf/Ppbp/Cxcl13/Bmp3/Bmp7/Inhba/Pgf/Tnf/Ccl11/Ccl3/Prl2a1/Pf4/Ccl8/Cxcl12/Angpt1/Efna5/Il6st/Tgfb1/Cxcl16/Tgfbr1/Il34/Ccl4/Angpt2/Il10/Bmp5/Gata3/Itgb3/Cntfr/Clcf1/Bmp8b/Tnfsf9/Ccl21a/Rasl11b
GO:0002523	leukocyte migration involved in inflammatory response	1.06E-07	10	S100a8/S100a9/Ccl2/Itgam/Ppbp/Adam8/Itgb2/Lbp/Slamf8/Jam3
GO:0070665	positive regulation of leukocyte proliferation	1.58E-07	29	Il6/Il1b/Tacr1/Vcam1/Igf1/Ager/Il6st/Havcr2/Cd4/Tac1/Cd80/Foxp3/Ccdc88b/Il2ra/Cd244a/Ptpn22/Ccr7/Cd86/Ccr2/Bst1/Ocstamp/Aif1/Clcf1/Coro1a/Cd6/Tnfsf9/Nckap1l/Tnfrsf4/Vtcn1
GO:0070555	response to interleukin-1	1.68E-07	22	Il6/Saa3/Serpine1/Il1r2/Il1r1/Ccl2/Sele/Il1a/Ccl11/Cebpb/Ccl3/Acod1/Ccl8/Adamts7/Fn1/Zc3h12a/Egr1/Ccl4/Rora/Irak3/Adamts12/Ccl21a
GO:0042102	positive regulation of T cell proliferation	1.85E-07	23	Il6/Il1b/Vcam1/Igf1/Ager/Il6st/Havcr2/Cd4/Cd80/Foxp3/Ccdc88b/Il2ra/Cd244a/Ptpn22/Ccr7/Cd86/Ccr2/Aif1/Coro1a/Cd6/Tnfsf9/Nckap1l/Vtcn1
GO:0032695	negative regulation of interleukin-12 production	2.04E-07	10	Thbs1/Tnfrsf9/Mefv/Arrb2/Cmklr1/Il10/Tlr2/Tlr8/Tigit/Irak3
GO:2000514	regulation of CD4-positive, alpha-beta T cell activation	2.09E-07	18	Il6/Nlrp3/Tarm1/Arg2/Zc3h12a/Hlx/Ager/Vsir/Tgfb1/Il4ra/Runx1/Foxp3/Ccr7/Myb/Ccr2/Gata3/Nckap1l/Runx3
GO:0002819	regulation of adaptive immune response	2.98E-07	35	Il1rl1/Il6/Il1b/Il1r1/C3/Fcgr3/Fcgr2b/Fcer1g/Il33/Nlrp3/Tnf/Arg1/Fcgr1/Slc11a1/Zc3h12a/Hlx/Ager/Samsn1/Vsir/Tgfb1/Alox15/Havcr2/Il4ra/Cd4/Arid5a/Foxp3/Cd55/Ccr7/H2-Q10/Ccr2/Gata3/Il7r/Clcf1/Il18r1/Il20rb
GO:0032651	regulation of interleukin-1 beta production	3.15E-07	17	Sphk1/Nlrp3/Arg2/Ccl3/Zc3h12a/Mefv/Hspb1/Egr1/Arrb2/Wnt5a/Ccr7/F2r/Apoa1/Tlr2/Ccr5/Tlr8/S1pr3
GO:0001776	leukocyte homeostasis	3.31E-07	24	Cxcl5/Il6/Cxcr2/Ccl2/Fcgr2b/Fcer1g/Pirb/Pde4b/Tgfb1/Axl/Spns2/Bcl2a1a/Foxp3/Ccnb2/Il2ra/Ccr2/Hcar2/Sh2b2/Slc7a11/Coro1a/Nckap1l/Nkx2-3/Tnfrsf4/Jam3
GO:0045582	positive regulation of T cell differentiation	4.34E-07	22	Il6/Il1b/Nlrp3/Vnn1/Adam8/Hlx/Vsir/Tgfb1/Il4ra/Runx1/Foxp3/Il2ra/Zbtb16/Ccr7/Myb/Ccr2/Pik3r6/Gata3/Il7r/Tnfsf9/Nckap1l/Runx3
GO:0002701	negative regulation of production of molecular mediator of immune response	4.37E-07	13	Fcgr2b/Hmox1/Il33/Tnf/Arg1/Angpt1/Vsir/Tgfb1/Foxp3/Il10/Il13ra2/Apoa1/Irak3
GO:0061756	leukocyte adhesion to vascular endothelial cell	4.95E-07	12	Selp/Sele/Vcam1/Tnf/Cxcl12/Ets1/Ptafr/Madcam1/Icam1/Sell/Ccr2/Selplg
GO:0002822	regulation of adaptive immune response based on somatic recombination of immune receptors built from immunoglobulin superfamily domains	4.95E-07	33	Il1rl1/Il6/Il1b/Il1r1/C3/Fcgr3/Fcgr2b/Fcer1g/Il33/Nlrp3/Tnf/Arg1/Fcgr1/Slc11a1/Zc3h12a/Hlx/Ager/Vsir/Tgfb1/Havcr2/Il4ra/Cd4/Arid5a/Foxp3/Cd55/Ccr7/H2-Q10/Ccr2/Gata3/Il7r/Clcf1/Il18r1/Il20rb
GO:0002460	adaptive immune response based on somatic recombination of immune receptors built from immunoglobulin superfamily domains	5.73E-07	57	Il1rl1/Il6/Il1b/Il1r1/C3/Fcgr3/Fcgr2b/C1qa/Fcer1g/Il33/Nlrp3/Tnf/C1s1/Arg1/Pirb/Fcgr1/C4b/C1qb/Slc11a1/Serping1/Il18rap/Zc3h12a/C1qc/Hlx/Ager/Vsir/Ighv1-72/Tgfb1/Havcr2/Il4ra/C1ra/Cd4/Arid5a/Stx11/Batf/Foxp3/Nras/Icam1/Rftn1/Cd55/Il13ra2/Ccr7/Ighg2c/C1rb/H2-Q10/Ccr2/Rora/Trdc/Gata3/Il7r/Clcf1/Il18r1/Ighv1-55/Il20rb/Inpp5d/Exo1/C3ar1
GO:0033004	negative regulation of mast cell activation	6.70E-07	8	Hmox1/Cd300lf/Foxf1/Cd300a/Cd84/Il13ra2/Milr1/Cnr1
GO:0032652	regulation of interleukin-1 production	6.80E-07	19	Il1r2/Sphk1/Nlrp3/Arg2/Ccl3/Zc3h12a/Mefv/Hspb1/Egr1/Arrb2/Havcr2/Wnt5a/Ccr7/F2r/Apoa1/Tlr2/Ccr5/Tlr8/S1pr3
GO:0002825	regulation of T-helper 1 type immune response	7.44E-07	12	Il1rl1/Il1b/Il1r1/Il33/Slc11a1/Hlx/Havcr2/Il4ra/Arid5a/Ccr7/Ccr2/Il18r1
GO:0045580	regulation of T cell differentiation	7.87E-07	28	Il6/Il1b/Ctla2a/Nlrp3/Ctla4/Vnn1/Adam8/Zc3h12a/Hlx/Vsir/Tgfb1/Il4ra/Runx1/Foxp3/Il2ra/Zbtb16/Ccr7/Myb/Ccr2/Zeb1/Pik3r6/Gata3/Il7r/Prdm1/Fanca/Tnfsf9/Nckap1l/Runx3
GO:0002704	negative regulation of leukocyte mediated immunity	7.88E-07	16	Fcgr2b/Hmox1/Foxf1/Arg1/Vsir/Cd300a/Arrb2/Havcr2/Cd84/Foxp3/Il13ra2/Ccr2/Il7r/Sh2d1b1/Il20rb/Klrb1b
GO:0002761	regulation of myeloid leukocyte differentiation	8.51E-07	25	Tnfsf11/Sfrp1/Rbp1/Tnf/Ccr1/Tyrobp/Cebpb/Ccl3/Pf4/Adam8/C1qc/Tgfb1/Cd4/Il34/Runx1/Csf1r/Hcls1/Fgfr3/Itgb3/Zfp36l1/Gata2/Ocstamp/Fbn1/Tal1/Inpp5d
GO:0045576	mast cell activation	8.62E-07	18	Fcgr3/Hmox1/Fcer1g/Cd300lf/Foxf1/Cd300a/Il4ra/Pla2g3/Cd84/Il13ra2/Cd300lb/Rac2/Milr1/Lcp2/Cnr1/Gata2/Nr4a3/Fgr
GO:0002720	positive regulation of cytokine production involved in immune response	8.74E-07	15	Il6/Il1b/Il1r1/Sema7a/Fcer1g/Nlrp3/Cd36/Gprc5b/Arid5a/Wnt5a/Tlr2/Gata3/Spon2/Il18r1/Nr4a3
GO:0002702	positive regulation of production of molecular mediator of immune response	8.74E-07	22	Il6/Il1b/Il1r1/Sema7a/Fcer1g/Il33/Nlrp3/Cd36/Gprc5b/Tgfb1/Il4ra/Arid5a/Wnt5a/Cd244a/Ptpn22/Tlr2/Gata3/Spon2/Clcf1/Il18r1/Nr4a3/Tnfrsf4
GO:0033003	regulation of mast cell activation	1.15E-06	15	Hmox1/Fcer1g/Cd300lf/Foxf1/Cd300a/Il4ra/Cd84/Il13ra2/Cd300lb/Rac2/Milr1/Cnr1/Gata2/Nr4a3/Fgr
GO:0010935	regulation of macrophage cytokine production	1.49E-06	8	Sema7a/Cd36/Gprc5b/Tgfb1/Wnt5a/Tlr2/Irak3/Spon2
GO:0045088	regulation of innate immune response	1.50E-06	39	Ereg/Mmp12/Mmp2/Cd300lf/Ltf/Tlr13/Tnf/Colec12/Arg1/Ccr1/Acod1/Serping1/Adam8/Il18rap/Cd36/Cd300a/Arrb2/Havcr2/A2m/Wnt5a/Rftn1/Ptpn22/Cav1/Cd86/Card9/Tlr2/Tlr8/Tlr7/Pik3r6/Irak3/Cadm1/Lbp/Lag3/Sh2d1b1/Slamf8/Pik3ap1/Klrb1b/Fgr/Myo1f
GO:0042108	positive regulation of cytokine biosynthetic process	2.12E-06	17	Thbs1/Ereg/Il1b/Fcgr3/Il1a/Tnf/Ptafr/Hspb1/Egr1/Wnt5a/Rel/Ccr2/Tlr2/Tlr8/Tlr7/Lbp/Cybb
GO:0043367	CD4-positive, alpha-beta T cell differentiation	2.25E-06	19	Il6/Nlrp3/Zc3h12a/Hlx/Tgfb1/Il4ra/Runx1/Batf/Foxp3/Ccr7/Satb1/Myb/Ccr2/Rora/Gata3/Il18r1/Nckap1l/Runx3/Nkx2-3
GO:0032655	regulation of interleukin-12 production	2.56E-06	15	Thbs1/Tnfrsf9/Cd36/Mefv/Ager/Arrb2/Cmklr1/Rel/Il10/Ccr7/Tlr2/Tlr8/Tigit/Irak3/Tnfsf9
GO:0061082	myeloid leukocyte cytokine production	2.75E-06	11	Sema7a/Hmox1/Fcer1g/Cd36/Gprc5b/Tgfb1/Wnt5a/Tlr2/Irak3/Spon2/Nr4a3
GO:0034446	substrate adhesion-dependent cell spreading	2.83E-06	20	Lamb1/Lamc1/Pdpn/Sfrp1/Ephb3/Antxr1/Fn1/Dab2/Prex1/Efna5/Axl/Fzd4/Fndc3b/Lama5/Parvb/Apoa1/Itgb3/Dbn1/Fermt3/Fbln1
GO:0002719	negative regulation of cytokine production involved in immune response	2.97E-06	10	Hmox1/Tnf/Arg1/Angpt1/Vsir/Tgfb1/Foxp3/Il10/Apoa1/Irak3
GO:0070486	leukocyte aggregation	3.04E-06	8	S100a8/S100a9/Il1b/Msn/Bmp7/Adam8/Rac2/Nr4a3
GO:0072678	T cell migration	3.23E-06	15	Apod/Ccl2/Cxcl13/S1pr1/Msn/Adam8/Cxcl12/Cxcl16/Wnt5a/Icam1/Ccr7/Ccr2/Itgb3/Aif1/Ccl21a
GO:0043300	regulation of leukocyte degranulation	3.82E-06	14	Hmox1/Fcer1g/Itgam/Foxf1/Ptafr/Cd300a/Il4ra/Cd84/Itgb2/Il13ra2/Ccr2/Rac2/Gata2/Fgr
GO:0042088	T-helper 1 type immune response	3.82E-06	14	Il1rl1/Il1b/Il1r1/Il33/Slc11a1/Il18rap/Hlx/Havcr2/Il4ra/Arid5a/Nras/Ccr7/Ccr2/Il18r1
GO:0002253	activation of immune response	4.30E-06	60	Fpr2/Fpr1/C3/Fcgr2b/C1qa/Fcer1g/Cd300lf/Ltf/Tlr13/Tnf/Ctla4/Colec12/C1s1/C4b/C1qb/Pde4b/Acod1/Serping1/Cd36/Zc3h12a/Cfh/C1qc/Ighv1-72/Cd300a/Arrb2/Havcr2/C1ra/Cmtm3/Cmklr1/A2m/Bcl2a1d/Foxp3/Nras/Rftn1/Cd55/Ptpn22/Cav1/Cfp/Ccr7/Ighg2c/Cd86/C1rb/Fcna/Tlr2/Trdc/Tlr8/Tlr7/Gata3/Irak3/Sh2b2/Lbp/Lcp2/Fyn/Nr4a3/Ighv1-55/Nckap1l/Pik3ap1/C6/Vtcn1/C3ar1
GO:0002292	T cell differentiation involved in immune response	4.79E-06	17	Il6/Clec4e/Fcer1g/Nlrp3/Clec4d/Zc3h12a/Hlx/Tgfb1/Il4ra/Batf/Foxp3/Ccr7/Myb/Ccr2/Rora/Gata3/Il18r1
GO:0002448	mast cell mediated immunity	4.89E-06	14	Hmox1/Fcer1g/Foxf1/Cd300a/Il4ra/Pla2g3/Cd84/Il13ra2/Rac2/Milr1/Spon2/Gata2/Nr4a3/Fgr
GO:0032615	interleukin-12 production	5.17E-06	15	Thbs1/Tnfrsf9/Cd36/Mefv/Ager/Arrb2/Cmklr1/Rel/Il10/Ccr7/Tlr2/Tlr8/Tigit/Irak3/Tnfsf9
GO:0002820	negative regulation of adaptive immune response	5.59E-06	13	Il1rl1/Fcgr2b/Il33/Arg1/Zc3h12a/Samsn1/Vsir/Alox15/Havcr2/Il4ra/Foxp3/Il7r/Il20rb
GO:0010934	macrophage cytokine production	5.67E-06	8	Sema7a/Cd36/Gprc5b/Tgfb1/Wnt5a/Tlr2/Irak3/Spon2
GO:0043370	regulation of CD4-positive, alpha-beta T cell differentiation	8.07E-06	14	Il6/Nlrp3/Zc3h12a/Hlx/Tgfb1/Il4ra/Runx1/Foxp3/Ccr7/Myb/Ccr2/Gata3/Nckap1l/Runx3
GO:0002369	T cell cytokine production	8.07E-06	12	Il6/Il1b/Il1r1/Nlrp3/Arg1/Slc11a1/Il18rap/Vsir/Arid5a/Foxp3/Gata3/Il18r1
GO:0050901	leukocyte tethering or rolling	1.01E-05	10	Selp/Sele/Vcam1/Tnf/Cxcl12/Ptafr/Madcam1/Sell/Ccr2/Selplg
GO:0032653	regulation of interleukin-10 production	1.02E-05	14	Mmp8/Tnfrsf9/Fcgr2b/Fcer1g/Ager/Vsir/Cd84/Foxp3/Tlr2/Tigit/Hgf/Trib2/Il20rb/Cd34
GO:0002440	production of molecular mediator of immune response	1.08E-05	44	Il6/Il1b/Il1r1/Sema7a/Trem1/Fcgr2b/Hmox1/Fcer1g/Il33/Nlrp3/Tnf/Arg1/Slc11a1/Igkv3-2/Il18rap/Cd36/Gprc5b/Angpt1/Vsir/Tgfb1/Il4ra/Arid5a/Batf/Foxp3/Wnt5a/Il10/Itm2a/Cd244a/Il13ra2/Ptpn22/Apoa1/Tlr2/Gata3/Irak3/Il7r/Igkv12-89/Spon2/Clcf1/Il18r1/Nr4a3/Polq/Igkv10-96/Tnfrsf4/Exo1
GO:0042226	interleukin-6 biosynthetic process	1.11E-05	9	Ereg/Il1b/Il19/Cebpb/Ptafr/Prg4/Muc16/Card9/Inpp5d
GO:0032623	interleukin-2 production	1.14E-05	16	Il1b/Il1a/Fcer1g/Slc11a1/Pde4b/Havcr2/Runx1/Foxp3/Ccr2/Card9/Gata3/Lag3/Nav3/Il20rb/Vtcn1/Cd34
GO:0046635	positive regulation of alpha-beta T cell activation	1.40E-05	16	Il6/Nlrp3/Hlx/Hsph1/Il4ra/Runx1/Cd80/Foxp3/Zbtb16/Cd244a/Ptpn22/Ccr7/Myb/Ccr2/Nckap1l/Runx3
GO:0002724	regulation of T cell cytokine production	1.45E-05	10	Il6/Il1b/Il1r1/Nlrp3/Arg1/Vsir/Arid5a/Foxp3/Gata3/Il18r1
GO:0046637	regulation of alpha-beta T cell differentiation	1.68E-05	16	Il6/Nlrp3/Zc3h12a/Hlx/Tgfb1/Il4ra/Runx1/Foxp3/Zbtb16/Ccr7/Myb/Ccr2/Gata3/Prdm1/Nckap1l/Runx3
GO:0061081	positive regulation of myeloid leukocyte cytokine production involved in immune response	1.69E-05	8	Sema7a/Fcer1g/Cd36/Gprc5b/Wnt5a/Tlr2/Spon2/Nr4a3
GO:0035743	CD4-positive, alpha-beta T cell cytokine production	1.70E-05	9	Il6/Il1b/Il1r1/Nlrp3/Arg1/Il18rap/Arid5a/Gata3/Il18r1
GO:2000106	regulation of leukocyte apoptotic process	1.71E-05	21	Cxcr2/Fcgr2b/Fcer1g/Arg2/Adam8/Cxcl12/Axl/Bcl2a1a/Wnt5a/Il10/Hcls1/Ccr7/Ccr5/Hcar2/Pdcd1/Il7r/Aurkb/Slc7a11/Nr4a3/Siglec1/Tnfrsf4
GO:0002286	T cell activation involved in immune response	1.71E-05	21	Il6/Clec4e/Fcer1g/Nlrp3/Clec4d/Slc11a1/Zc3h12a/Hlx/Tgfb1/Havcr2/Il4ra/Stx11/Batf/Foxp3/Icam1/Ccr7/Myb/Ccr2/Rora/Gata3/Il18r1
GO:0032613	interleukin-10 production	1.98E-05	14	Mmp8/Tnfrsf9/Fcgr2b/Fcer1g/Ager/Vsir/Cd84/Foxp3/Tlr2/Tigit/Hgf/Trib2/Il20rb/Cd34
GO:0043303	mast cell degranulation	1.98E-05	13	Hmox1/Fcer1g/Foxf1/Cd300a/Il4ra/Pla2g3/Cd84/Il13ra2/Rac2/Milr1/Gata2/Nr4a3/Fgr
GO:0002920	regulation of humoral immune response	2.00E-05	11	C3/Fcgr2b/Tnf/Acod1/Serping1/Cfh/Spns2/A2m/Cd55/Ccr7/C6
GO:0046632	alpha-beta T cell differentiation	2.21E-05	21	Il6/Nlrp3/Zc3h12a/Hlx/Tgfb1/Il4ra/Runx1/Batf/Foxp3/Zbtb16/Ccr7/Satb1/Myb/Ccr2/Rora/Gata3/Prdm1/Il18r1/Nckap1l/Runx3/Nkx2-3
GO:0002705	positive regulation of leukocyte mediated immunity	2.26E-05	28	Il6/Il1b/Cxcl1/Il1r1/C3/Fcgr3/Fcer1g/Itgam/Nlrp3/Tnf/Arg1/Fcgr1/Il18rap/Ptafr/Tgfb1/Il4ra/Arid5a/Itgb2/Foxp3/H2-Q10/Gata3/Cadm1/Lag3/Sh2d1b1/Gata2/Clcf1/Il18r1/Fgr
GO:0050702	interleukin-1 beta secretion	2.36E-05	12	Abca1/Nlrp3/Arg2/Ccl3/Cd36/Zc3h12a/Wnt5a/Ccr7/Apoa1/Tlr2/Ccr5/Tlr8
GO:0042130	negative regulation of T cell proliferation	2.37E-05	16	Lrrc32/Tarm1/Ctla4/Arg2/Arg1/Gpnmb/Cebpb/Vsir/Tgfb1/Havcr2/Cd80/Foxp3/Il2ra/Cd86/Il20rb/Vtcn1
GO:0002279	mast cell activation involved in immune response	2.44E-05	13	Hmox1/Fcer1g/Foxf1/Cd300a/Il4ra/Pla2g3/Cd84/Il13ra2/Rac2/Milr1/Gata2/Nr4a3/Fgr
GO:0071887	leukocyte apoptotic process	2.85E-05	23	Il6/Cxcr2/Fcgr2b/Fcer1g/Arg2/Adam8/Cxcl12/Axl/Bcl2a1a/Wnt5a/Il10/Il2ra/Hcls1/Ccr7/Ccr5/Hcar2/Pdcd1/Il7r/Aurkb/Slc7a11/Nr4a3/Siglec1/Tnfrsf4
GO:0050701	interleukin-1 secretion	3.05E-05	13	Il1r2/Abca1/Nlrp3/Arg2/Ccl3/Cd36/Zc3h12a/Wnt5a/Ccr7/Apoa1/Tlr2/Ccr5/Tlr8
GO:0042092	type 2 immune response	3.48E-05	11	Il6/Il33/Nlrp3/Arg2/Arg1/Hlx/Il4ra/Batf/Ccr2/Gata3/Clcf1
GO:0002828	regulation of type 2 immune response	3.75E-05	10	Il6/Il33/Nlrp3/Arg2/Arg1/Hlx/Il4ra/Ccr2/Gata3/Clcf1
GO:0002888	positive regulation of myeloid leukocyte mediated immunity	3.79E-05	12	Cxcl1/C3/Fcgr3/Fcer1g/Itgam/Arg1/Fcgr1/Ptafr/Il4ra/Itgb2/Gata2/Fgr
GO:0032663	regulation of interleukin-2 production	4.33E-05	14	Il1b/Il1a/Pde4b/Havcr2/Runx1/Foxp3/Ccr2/Card9/Gata3/Lag3/Nav3/Il20rb/Vtcn1/Cd34
GO:0043301	negative regulation of leukocyte degranulation	4.39E-05	6	Hmox1/Foxf1/Cd300a/Cd84/Il13ra2/Ccr2
GO:0072604	interleukin-6 secretion	4.52E-05	11	Il1b/Zc3h12a/Nos2/Arid5a/Cd84/Ptpn22/F2r/Trpv4/Twist1/Tlr8/Syt11
GO:0032637	interleukin-8 production	5.48E-05	15	Serpine1/Il1b/Chil1/Tnf/Nos2/Wnt5a/Afap1l2/Cd244a/Ptpn22/F2r/Tlr2/Tlr8/Tlr7/Ssc5d/Lbp
GO:0032693	negative regulation of interleukin-10 production	6.12E-05	8	Mmp8/Tnfrsf9/Fcgr2b/Ager/Vsir/Cd84/Foxp3/Trib2
GO:0045408	regulation of interleukin-6 biosynthetic process	6.12E-05	8	Ereg/Il1b/Cebpb/Ptafr/Prg4/Muc16/Card9/Inpp5d
GO:0002374	cytokine secretion involved in immune response	6.12E-05	8	Trem1/Nlrp3/Tnf/Angpt1/Wnt5a/Il10/Apoa1/Tlr2
GO:0002763	positive regulation of myeloid leukocyte differentiation	6.24E-05	14	Tnfsf11/Tnf/Ccr1/Ccl3/Pf4/Tgfb1/Cd4/Il34/Runx1/Csf1r/Hcls1/Itgb3/Zfp36l1/Ocstamp
GO:0002823	negative regulation of adaptive immune response based on somatic recombination of immune receptors built from immunoglobulin superfamily domains	7.33E-05	11	Il1rl1/Fcgr2b/Il33/Arg1/Zc3h12a/Vsir/Havcr2/Il4ra/Foxp3/Il7r/Il20rb
GO:0002283	neutrophil activation involved in immune response	0.000100258	7	Fcer1g/Itgam/Tyrobp/Ptafr/Stx11/Itgb2/Myo1f
GO:0045089	positive regulation of innate immune response	0.000100884	30	Ereg/Mmp12/Mmp2/Cd300lf/Ltf/Tlr13/Tnf/Colec12/Acod1/Adam8/Il18rap/Cd36/Cd300a/Arrb2/Havcr2/Wnt5a/Rftn1/Ptpn22/Cav1/Cd86/Card9/Tlr2/Tlr8/Tlr7/Irak3/Cadm1/Lbp/Lag3/Sh2d1b1/Pik3ap1
GO:0002294	CD4-positive, alpha-beta T cell differentiation involved in immune response	0.000105367	14	Il6/Nlrp3/Zc3h12a/Hlx/Tgfb1/Il4ra/Batf/Foxp3/Ccr7/Myb/Ccr2/Rora/Gata3/Il18r1
GO:0043304	regulation of mast cell degranulation	0.000112252	10	Hmox1/Fcer1g/Foxf1/Cd300a/Il4ra/Cd84/Il13ra2/Rac2/Gata2/Fgr
GO:0002293	alpha-beta T cell differentiation involved in immune response	0.000124943	14	Il6/Nlrp3/Zc3h12a/Hlx/Tgfb1/Il4ra/Batf/Foxp3/Ccr7/Myb/Ccr2/Rora/Gata3/Il18r1
GO:0046638	positive regulation of alpha-beta T cell differentiation	0.00013564	12	Il6/Nlrp3/Hlx/Il4ra/Runx1/Foxp3/Zbtb16/Ccr7/Myb/Ccr2/Nckap1l/Runx3
GO:0002285	lymphocyte activation involved in immune response	0.000140936	27	Il6/Clec4e/Fcer1g/Nlrp3/Clec4d/Slc11a1/Zc3h12a/Hlx/Tgfb1/Havcr2/Il4ra/Stx11/Batf/Foxp3/Icam1/Itm2a/Cd244a/Ccr7/Myb/Ccr2/Rora/Gata3/Clcf1/Il18r1/Coro1a/Nkx2-3/Exo1
GO:0050706	regulation of interleukin-1 beta secretion	0.000143647	10	Nlrp3/Arg2/Ccl3/Zc3h12a/Wnt5a/Ccr7/Apoa1/Tlr2/Ccr5/Tlr8
GO:0033006	regulation of mast cell activation involved in immune response	0.000143647	10	Hmox1/Fcer1g/Foxf1/Cd300a/Il4ra/Cd84/Il13ra2/Rac2/Gata2/Fgr
GO:0050704	regulation of interleukin-1 secretion	0.000144365	11	Il1r2/Nlrp3/Arg2/Ccl3/Zc3h12a/Wnt5a/Ccr7/Apoa1/Tlr2/Ccr5/Tlr8
GO:0046636	negative regulation of alpha-beta T cell activation	0.000144365	11	Tarm1/Arg2/Zc3h12a/Hlx/Vsir/Cd300a/Il4ra/Runx1/Foxp3/Adora2a/Runx3
GO:0002287	alpha-beta T cell activation involved in immune response	0.000145765	14	Il6/Nlrp3/Zc3h12a/Hlx/Tgfb1/Il4ra/Batf/Foxp3/Ccr7/Myb/Ccr2/Rora/Gata3/Il18r1
GO:1904994	regulation of leukocyte adhesion to vascular endothelial cell	0.000145956	6	Tnf/Cxcl12/Ets1/Ptafr/Icam1/Ccr2
GO:0042036	negative regulation of cytokine biosynthetic process	0.000165715	9	Il6/Inhbb/Prg4/Muc16/Foxp3/Il10/Lag3/Trib2/Inpp5d
GO:0002827	positive regulation of T-helper 1 type immune response	0.000175032	8	Il1b/Il1r1/Slc11a1/Hlx/Arid5a/Ccr7/Ccr2/Il18r1
GO:0043372	positive regulation of CD4-positive, alpha-beta T cell differentiation	0.000218687	9	Il6/Nlrp3/Hlx/Il4ra/Foxp3/Ccr7/Myb/Ccr2/Nckap1l
GO:0032757	positive regulation of interleukin-8 production	0.000219443	11	Serpine1/Il1b/Tnf/Wnt5a/Afap1l2/Cd244a/F2r/Tlr2/Tlr8/Tlr7/Lbp
GO:0002726	positive regulation of T cell cytokine production	0.000224164	7	Il6/Il1b/Il1r1/Nlrp3/Arid5a/Gata3/Il18r1
GO:0032753	positive regulation of interleukin-4 production	0.000237696	8	Fcer1g/Il33/Nlrp3/Cebpb/Havcr2/Foxp3/Gata3/Il20rb
GO:2000515	negative regulation of CD4-positive, alpha-beta T cell activation	0.000279683	9	Tarm1/Arg2/Zc3h12a/Hlx/Vsir/Il4ra/Runx1/Foxp3/Runx3
GO:0032677	regulation of interleukin-8 production	0.000281381	13	Serpine1/Il1b/Tnf/Wnt5a/Afap1l2/Cd244a/Ptpn22/F2r/Tlr2/Tlr8/Tlr7/Ssc5d/Lbp
GO:0006959	humoral immune response	0.000281381	41	S100a9/Cxcl5/Cxcl2/Cxcl1/Cxcl3/Ccl2/C3/Fcgr2b/Fga/C1qa/Ppbp/Cxcl13/Ltf/Tnf/C1s1/C4b/C1qb/Acod1/Serping1/Pf4/Cfh/C1qc/Ighv1-72/Spns2/C1ra/A2m/Cd55/Cfp/Ccr7/Rarres2/Ighg2c/C1rb/Fcna/Ccr2/Trdc/Gata3/Slpi/Spon2/Ighv1-55/C6/Exo1
GO:0045824	negative regulation of innate immune response	0.000281381	12	Mmp12/Arg1/Ccr1/Acod1/Serping1/Arrb2/Havcr2/A2m/Irak3/Sh2d1b1/Slamf8/Klrb1b
GO:0002686	negative regulation of leukocyte migration	0.000282957	10	Apod/Grem1/Dusp1/Nbl1/Il33/Cxcl12/Emilin1/Cd300a/Adora1/Slamf8
GO:0002764	immune response-regulating signaling pathway	0.000291533	49	Fpr2/Fpr1/Clec4e/Fcgr2b/Fcer1g/Cd300lf/Ltf/Tlr13/Tnf/Ctla4/Colec12/Clec4d/Pde4b/Acod1/Cd36/Zc3h12a/Ighv1-72/Cd300a/Arrb2/Havcr2/Cmtm3/Cmklr1/Bcl2a1d/Foxp3/Nras/Rftn1/Ptpn22/Cav1/Ccr7/Ighg2c/Cd86/Tlr2/Trdc/Tlr8/Tlr7/Gata3/Irak3/Sh2b2/Lbp/Lcp2/Fyn/Sh2d1b1/Nr4a3/Ighv1-55/Nckap1l/Pik3ap1/Il20rb/Vtcn1/C3ar1
GO:0072606	interleukin-8 secretion	0.000318798	8	Chil1/Nos2/Wnt5a/Cd244a/Ptpn22/F2r/Tlr2/Ssc5d
GO:0050798	activated T cell proliferation	0.000320711	11	Itgam/Lrrc32/Arg1/Igf1/Ager/Itgb2/Il2ra/Satb1/Cd86/Fyn/Tnfsf9
GO:1900015	regulation of cytokine production involved in inflammatory response	0.000349981	10	Il1r2/Apod/Per1/Tarm1/Zc3h12a/Mefv/Nos2/Sirpa/Gpsm3/Cd6
GO:0050869	negative regulation of B cell activation	0.00035374	9	Fcgr2b/Sfrp1/Ctla4/Samsn1/Cd300a/Foxp3/Il10/Prdm1/Inpp5d
GO:0002758	innate immune response-activating signal transduction	0.000423651	20	Cd300lf/Ltf/Tlr13/Tnf/Colec12/Acod1/Cd36/Cd300a/Arrb2/Havcr2/Rftn1/Ptpn22/Cav1/Cd86/Tlr2/Tlr8/Tlr7/Irak3/Lbp/Pik3ap1
GO:0001909	leukocyte mediated cytotoxicity	0.00050804	21	Cxcl5/Cxcl1/Ccl2/Trem1/Fcgr3/Arg1/Fcgr1/Il18rap/Ager/Arrb2/Havcr2/Stx11/H2-Q10/Pik3r6/Cadm1/Lag3/Il7r/Sh2d1b1/Coro1a/Klrb1b/Ncf1
GO:2000404	regulation of T cell migration	0.000510501	10	Apod/Cxcl13/Adam8/Cxcl12/Wnt5a/Ccr7/Ccr2/Itgb3/Aif1/Ccl21a
GO:0014033	neural crest cell differentiation	0.00052764	16	Sema7a/Sfrp1/Ednra/Bmp7/Rdh10/Fn1/Ret/Sema3f/Ednrb/Lama5/Sema6b/Twist1/Hand2/Snai2/Phox2b/Sema3g
GO:0036037	CD8-positive, alpha-beta T cell activation	0.000530507	8	Vsir/Runx1/Cd244a/Ptpn22/Satb1/Clec4a2/Nckap1l/Runx3
GO:2000406	positive regulation of T cell migration	0.000542479	9	Cxcl13/Adam8/Cxcl12/Wnt5a/Ccr7/Ccr2/Itgb3/Aif1/Ccl21a
GO:2000516	positive regulation of CD4-positive, alpha-beta T cell activation	0.000542479	9	Il6/Nlrp3/Hlx/Il4ra/Foxp3/Ccr7/Myb/Ccr2/Nckap1l
GO:0002830	positive regulation of type 2 immune response	0.000545532	6	Il6/Il33/Nlrp3/Il4ra/Gata3/Clcf1
GO:0002824	positive regulation of adaptive immune response based on somatic recombination of immune receptors built from immunoglobulin superfamily domains	0.000549094	21	Il6/Il1b/Il1r1/C3/Fcgr3/Fcer1g/Nlrp3/Tnf/Fcgr1/Slc11a1/Hlx/Tgfb1/Cd4/Arid5a/Foxp3/Ccr7/H2-Q10/Ccr2/Gata3/Clcf1/Il18r1
GO:0035744	T-helper 1 cell cytokine production	0.000550232	5	Il1b/Il1r1/Il18rap/Arid5a/Il18r1
GO:0002534	cytokine production involved in inflammatory response	0.000604767	10	Il1r2/Apod/Per1/Tarm1/Zc3h12a/Mefv/Nos2/Sirpa/Gpsm3/Cd6
GO:0006929	substrate-dependent cell migration	0.000668897	8	Adam8/Fn1/Fgfr1/Pdgfb/Tnfrsf12a/Snai2/P2ry12/Fbln1
GO:0014032	neural crest cell development	0.000668897	15	Sema7a/Ednra/Bmp7/Rdh10/Fn1/Ret/Sema3f/Ednrb/Lama5/Sema6b/Twist1/Hand2/Snai2/Phox2b/Sema3g
GO:0001755	neural crest cell migration	0.000706219	12	Sema7a/Bmp7/Fn1/Ret/Sema3f/Ednrb/Lama5/Sema6b/Twist1/Hand2/Phox2b/Sema3g
GO:0051852	disruption by host of symbiont cells	0.000777607	6	Cxcl5/Cxcl1/Trem1/Arg1/Pf4/Ncf1
GO:0051873	killing by host of symbiont cells	0.000777607	6	Cxcl5/Cxcl1/Trem1/Arg1/Pf4/Ncf1
GO:2000107	negative regulation of leukocyte apoptotic process	0.000849747	13	Cxcr2/Fcgr2b/Fcer1g/Arg2/Cxcl12/Axl/Bcl2a1a/Hcls1/Ccr7/Ccr5/Il7r/Aurkb/Tnfrsf4
GO:0070943	neutrophil mediated killing of symbiont cell	0.000878383	5	Cxcl5/Cxcl1/Trem1/Arg1/Ncf1
GO:0032620	interleukin-17 production	0.000977077	9	Osm/Arg2/Vsir/Tgfb1/Arid5a/Foxp3/Rftn1/Tlr2/Nckap1l
GO:0032633	interleukin-4 production	0.000977077	9	Fcer1g/Il33/Nlrp3/Cebpb/Havcr2/Foxp3/Gata3/Il20rb/Vtcn1
GO:0032731	positive regulation of interleukin-1 beta production	0.000977077	9	Nlrp3/Ccl3/Hspb1/Egr1/Wnt5a/Ccr7/Tlr2/Ccr5/Tlr8
GO:0002821	positive regulation of adaptive immune response	0.000978168	21	Il6/Il1b/Il1r1/C3/Fcgr3/Fcer1g/Nlrp3/Tnf/Fcgr1/Slc11a1/Hlx/Tgfb1/Cd4/Arid5a/Foxp3/Ccr7/H2-Q10/Ccr2/Gata3/Clcf1/Il18r1
GO:0032732	positive regulation of interleukin-1 production	0.001010452	10	Nlrp3/Ccl3/Hspb1/Egr1/Havcr2/Wnt5a/Ccr7/Tlr2/Ccr5/Tlr8
GO:0002269	leukocyte activation involved in inflammatory response	0.001029033	8	Mmp8/Fpr2/Il33/Ager/Tlr2/Tlr8/Tlr7/Aif1
GO:0032660	regulation of interleukin-17 production	0.001029033	8	Osm/Arg2/Vsir/Tgfb1/Arid5a/Foxp3/Tlr2/Nckap1l
GO:0032673	regulation of interleukin-4 production	0.001029033	8	Fcer1g/Il33/Nlrp3/Cebpb/Havcr2/Foxp3/Gata3/Il20rb
GO:0032700	negative regulation of interleukin-17 production	0.001060545	6	Arg2/Vsir/Tgfb1/Foxp3/Tlr2/Nckap1l
GO:2001185	regulation of CD8-positive, alpha-beta T cell activation	0.001060545	6	Vsir/Runx1/Cd244a/Ptpn22/Nckap1l/Runx3
GO:0001959	regulation of cytokine-mediated signaling pathway	0.001076097	16	Il6/Il1r2/Il1r1/Mmp12/Sphk1/Slit3/Cd300lf/Arg1/Trem2/Angpt1/Il6st/Axl/Wnt5a/Cav1/Apoa1/Irak3
GO:0060759	regulation of response to cytokine stimulus	0.001213528	17	Il6/Il1r2/Il1r1/Mmp12/Sphk1/Slit3/Cd300lf/Arg1/Trem2/Angpt1/Il6st/Axl/Wnt5a/Cav1/Apoa1/Tlr2/Irak3
GO:0072538	T-helper 17 type immune response	0.00127248	8	Il6/Nlrp3/Zc3h12a/Tgfb1/Arid5a/Batf/Foxp3/Rora
GO:0002757	immune response-activating signal transduction	0.001343103	45	Fpr2/Fpr1/Fcgr2b/Fcer1g/Cd300lf/Ltf/Tlr13/Tnf/Ctla4/Colec12/Pde4b/Acod1/Cd36/Zc3h12a/Ighv1-72/Cd300a/Arrb2/Havcr2/Cmtm3/Cmklr1/Bcl2a1d/Foxp3/Nras/Rftn1/Ptpn22/Cav1/Ccr7/Ighg2c/Cd86/Tlr2/Trdc/Tlr8/Tlr7/Gata3/Irak3/Sh2b2/Lbp/Lcp2/Fyn/Nr4a3/Ighv1-55/Nckap1l/Pik3ap1/Vtcn1/C3ar1
GO:0002456	T cell mediated immunity	0.001418302	19	Il6/Il1b/Il1r1/Nlrp3/Arg1/Slc11a1/Il18rap/Ager/Vsir/Arid5a/Stx11/Foxp3/Icam1/Rftn1/H2-Q10/Gata3/Il7r/Il18r1/Il20rb
GO:0032674	regulation of interleukin-5 production	0.001433829	6	Il1rl1/Il33/Nlrp3/Il5ra/Foxp3/Gata3
GO:0030889	negative regulation of B cell proliferation	0.001433829	6	Fcgr2b/Ctla4/Cd300a/Il10/Prdm1/Inpp5d
GO:0032703	negative regulation of interleukin-2 production	0.001607694	7	Havcr2/Foxp3/Gata3/Lag3/Nav3/Il20rb/Cd34
GO:0002218	activation of innate immune response	0.001884179	20	Cd300lf/Ltf/Tlr13/Tnf/Colec12/Acod1/Cd36/Cd300a/Arrb2/Havcr2/Rftn1/Ptpn22/Cav1/Cd86/Tlr2/Tlr8/Tlr7/Irak3/Lbp/Pik3ap1
GO:0032634	interleukin-5 production	0.001912936	6	Il1rl1/Il33/Nlrp3/Il5ra/Foxp3/Gata3
GO:0002517	T cell tolerance induction	0.001916359	5	Icos/Runx1/Foxp3/Il2ra/Cd86
GO:0002281	macrophage activation involved in immune response	0.001916359	5	Il33/Tyrobp/Havcr2/Lbp/Dysf
GO:0070498	interleukin-1-mediated signaling pathway	0.002486876	6	Il6/Il1r2/Il1r1/Il1a/Egr1/Irak3
GO:0006691	leukotriene metabolic process	0.003147625	6	Ggt5/Ggt1/Alox5ap/Cyp4f18/Tlr2/Ncf1
GO:2000482	regulation of interleukin-8 secretion	0.003147625	6	Wnt5a/Cd244a/Ptpn22/F2r/Tlr2/Ssc5d
GO:0002709	regulation of T cell mediated immunity	0.003296751	14	Il6/Il1b/Il1r1/Nlrp3/Arg1/Ager/Vsir/Arid5a/Foxp3/H2-Q10/Gata3/Il7r/Il18r1/Il20rb
GO:0033089	positive regulation of T cell differentiation in thymus	0.003628826	5	Il1b/Vnn1/Adam8/Foxp3/Il7r
GO:0002739	regulation of cytokine secretion involved in immune response	0.003628826	5	Tnf/Angpt1/Wnt5a/Il10/Apoa1
GO:1900016	negative regulation of cytokine production involved in inflammatory response	0.003628826	5	Il1r2/Apod/Zc3h12a/Mefv/Sirpa
GO:0043302	positive regulation of leukocyte degranulation	0.003637478	7	Fcer1g/Itgam/Ptafr/Il4ra/Itgb2/Gata2/Fgr
GO:0050718	positive regulation of interleukin-1 beta secretion	0.003637478	7	Nlrp3/Ccl3/Wnt5a/Ccr7/Tlr2/Ccr5/Tlr8
GO:0001960	negative regulation of cytokine-mediated signaling pathway	0.004611661	9	Il6/Il1r2/Mmp12/Slit3/Arg1/Il6st/Cav1/Apoa1/Irak3
GO:0032621	interleukin-18 production	0.004750601	4	Nlrp3/Tnf/Cd84/Tlr2
GO:0050711	negative regulation of interleukin-1 secretion	0.004750601	4	Il1r2/Nlrp3/Zc3h12a/Apoa1
GO:0070673	response to interleukin-18	0.004750601	4	Il18rap/Pdgfb/Cldn1/Il18r1
GO:0043371	negative regulation of CD4-positive, alpha-beta T cell differentiation	0.004760181	6	Zc3h12a/Hlx/Il4ra/Runx1/Foxp3/Runx3
GO:0019724	B cell mediated immunity	0.005107466	28	C3/Fcgr3/Fcgr2b/C1qa/Fcer1g/Tnf/C1s1/Pirb/Fcgr1/C4b/C1qb/Serping1/C1qc/Ighv1-72/Tgfb1/Il4ra/C1ra/Batf/Foxp3/Cd55/Il13ra2/Ighg2c/C1rb/Trdc/Clcf1/Ighv1-55/Inpp5d/Exo1
GO:0001910	regulation of leukocyte mediated cytotoxicity	0.005759764	15	Cxcl5/Cxcl1/Ccl2/Arg1/Il18rap/Ager/Arrb2/Havcr2/H2-Q10/Pik3r6/Cadm1/Lag3/Il7r/Sh2d1b1/Klrb1b
GO:0050716	positive regulation of interleukin-1 secretion	0.005990978	7	Nlrp3/Ccl3/Wnt5a/Ccr7/Tlr2/Ccr5/Tlr8
GO:0032691	negative regulation of interleukin-1 beta production	0.005990978	5	Nlrp3/Zc3h12a/Mefv/Arrb2/Apoa1
GO:0002922	positive regulation of humoral immune response	0.005990978	5	C3/Tnf/Acod1/Ccr7/C6
GO:1902106	negative regulation of leukocyte differentiation	0.006423457	14	Sfrp1/Tmem176a/Ctla4/Zc3h12a/C1qc/Hlx/Il4ra/Runx1/Foxp3/Tmem176b/Gata2/Fbn1/Runx3/Inpp5d
GO:0002664	regulation of T cell tolerance induction	0.006620994	4	Runx1/Foxp3/Il2ra/Cd86
GO:1901163	regulation of trophoblast cell migration	0.006620994	4	Timp1/Mmp12/Syde1/Arhgdib
GO:0032692	negative regulation of interleukin-1 production	0.006940312	6	Il1r2/Nlrp3/Zc3h12a/Mefv/Arrb2/Apoa1
GO:0060761	negative regulation of response to cytokine stimulus	0.007342352	9	Il6/Il1r2/Mmp12/Slit3/Arg1/Il6st/Cav1/Apoa1/Irak3
GO:0002710	negative regulation of T cell mediated immunity	0.007463658	5	Arg1/Vsir/Foxp3/Il7r/Il20rb
GO:2000108	positive regulation of leukocyte apoptotic process	0.008044325	7	Adam8/Wnt5a/Il10/Hcar2/Pdcd1/Nr4a3/Siglec1
GO:0016064	immunoglobulin mediated immune response	0.008070417	27	C3/Fcgr3/Fcgr2b/C1qa/Fcer1g/Tnf/C1s1/Fcgr1/C4b/C1qb/Serping1/C1qc/Ighv1-72/Tgfb1/Il4ra/C1ra/Batf/Foxp3/Cd55/Il13ra2/Ighg2c/C1rb/Trdc/Clcf1/Ighv1-55/Inpp5d/Exo1
GO:2000778	positive regulation of interleukin-6 secretion	0.008255899	6	Il1b/Arid5a/F2r/Trpv4/Twist1/Tlr8
GO:0045589	regulation of regulatory T cell differentiation	0.008255899	6	Ctla2a/Ctla4/Vsir/Tgfb1/Foxp3/Fanca
GO:0032754	positive regulation of interleukin-5 production	0.008895626	4	Il1rl1/Il33/Nlrp3/Gata3
GO:0061450	trophoblast cell migration	0.008895626	4	Timp1/Mmp12/Syde1/Arhgdib
GO:0046718	viral entry into host cell	0.009178108	9	Ifitm1/Ifitm6/Ptx3/Axl/Cd4/Cav1/Itgb3/Trim10/Trim21
GO:0045076	regulation of interleukin-2 biosynthetic process	0.009244491	5	Il1b/Il1a/Foxp3/Card9/Lag3
GO:0010818	T cell chemotaxis	0.009244491	5	Cxcl13/Cxcl16/Wnt5a/Ccr7/Ccr2
GO:0002712	regulation of B cell mediated immunity	0.009482699	10	C3/Fcgr3/Fcgr2b/Fcer1g/Tnf/Fcgr1/Tgfb1/Foxp3/Cd55/Clcf1
GO:0002889	regulation of immunoglobulin mediated immune response	0.009482699	10	C3/Fcgr3/Fcgr2b/Fcer1g/Tnf/Fcgr1/Tgfb1/Foxp3/Cd55/Clcf1
GO:0046639	negative regulation of alpha-beta T cell differentiation	0.009725406	6	Zc3h12a/Hlx/Il4ra/Runx1/Foxp3/Runx3
GO:0046640	regulation of alpha-beta T cell proliferation	0.010641684	7	Tarm1/Arg2/Vsir/Cd80/Cd244a/Ptpn22/Ccr2
GO:0042267	natural killer cell mediated cytotoxicity	0.011450593	10	Il18rap/Arrb2/Havcr2/Stx11/Pik3r6/Cadm1/Lag3/Sh2d1b1/Coro1a/Klrb1b
GO:2000484	positive regulation of interleukin-8 secretion	0.01148009	4	Wnt5a/Cd244a/F2r/Tlr2
GO:0035745	T-helper 2 cell cytokine production	0.01148009	4	Il6/Nlrp3/Arg1/Gata3
GO:0030260	entry into host cell	0.012367149	9	Ifitm1/Ifitm6/Ptx3/Axl/Cd4/Cav1/Itgb3/Trim10/Trim21
GO:0071353	cellular response to interleukin-4	0.01321804	6	Cd300lf/Nfil3/Mrc1/Gata3/Mcm2/Coro1a
GO:0033005	positive regulation of mast cell activation	0.01321804	6	Fcer1g/Il4ra/Cd300lb/Gata2/Nr4a3/Fgr
GO:0045066	regulatory T cell differentiation	0.01321804	6	Ctla2a/Ctla4/Vsir/Tgfb1/Foxp3/Fanca
GO:0042094	interleukin-2 biosynthetic process	0.01347299	5	Il1b/Il1a/Foxp3/Card9/Lag3
GO:0046006	regulation of activated T cell proliferation	0.013571162	7	Lrrc32/Arg1/Igf1/Ager/Il2ra/Cd86/Tnfsf9
GO:0002228	natural killer cell mediated immunity	0.013572012	10	Il18rap/Arrb2/Havcr2/Stx11/Pik3r6/Cadm1/Lag3/Sh2d1b1/Coro1a/Klrb1b
GO:0002768	immune response-regulating cell surface receptor signaling pathway	0.01370038	32	Fpr2/Fpr1/Clec4e/Fcgr2b/Fcer1g/Ctla4/Clec4d/Pde4b/Zc3h12a/Ighv1-72/Cd300a/Cmtm3/Cmklr1/Bcl2a1d/Foxp3/Nras/Rftn1/Ptpn22/Ccr7/Ighg2c/Tlr2/Trdc/Gata3/Sh2b2/Lcp2/Fyn/Sh2d1b1/Nr4a3/Ighv1-55/Nckap1l/Vtcn1/C3ar1
GO:0010820	positive regulation of T cell chemotaxis	0.014564028	4	Cxcl13/Wnt5a/Ccr7/Ccr2
GO:0002829	negative regulation of type 2 immune response	0.014564028	4	Arg2/Arg1/Hlx/Ccr2
GO:0030888	regulation of B cell proliferation	0.014756231	10	Fcgr2b/Ctla4/Cd300a/Il10/Bst1/Prdm1/Clcf1/Nckap1l/Tnfrsf4/Inpp5d
GO:0046633	alpha-beta T cell proliferation	0.015272178	7	Tarm1/Arg2/Vsir/Cd80/Cd244a/Ptpn22/Ccr2
GO:0001911	negative regulation of leukocyte mediated cytotoxicity	0.015904422	5	Arrb2/Havcr2/Il7r/Sh2d1b1/Klrb1b
GO:0070670	response to interleukin-4	0.017380764	6	Cd300lf/Nfil3/Mrc1/Gata3/Mcm2/Coro1a
GO:0033081	regulation of T cell differentiation in thymus	0.017380764	6	Il1b/Vnn1/Adam8/Foxp3/Zeb1/Il7r
GO:1900024	regulation of substrate adhesion-dependent cell spreading	0.018025241	8	Pdpn/Dab2/Prex1/Efna5/Apoa1/Itgb3/Dbn1/Fbln1
GO:0010819	regulation of T cell chemotaxis	0.018074986	4	Cxcl13/Wnt5a/Ccr7/Ccr2
GO:0070234	positive regulation of T cell apoptotic process	0.018074986	4	Adam8/Wnt5a/Pdcd1/Siglec1
GO:0032733	positive regulation of interleukin-10 production	0.019719796	6	Fcer1g/Tlr2/Tigit/Hgf/Il20rb/Cd34
GO:0032743	positive regulation of interleukin-2 production	0.019719796	6	Il1b/Il1a/Pde4b/Runx1/Ccr2/Vtcn1
GO:0045577	regulation of B cell differentiation	0.019719796	6	Sfrp1/Mmp14/Zfp36l1/Prdm1/Nckap1l/Inpp5d
GO:0043374	CD8-positive, alpha-beta T cell differentiation	0.022221339	4	Runx1/Satb1/Nckap1l/Runx3
GO:0046596	regulation of viral entry into host cell	0.022322781	6	Ifitm1/Ifitm6/Ptx3/Cd4/Trim10/Trim21
GO:0070232	regulation of T cell apoptotic process	0.023818542	7	Arg2/Adam8/Wnt5a/Pdcd1/Il7r/Siglec1/Tnfrsf4
GO:0042269	regulation of natural killer cell mediated cytotoxicity	0.023869632	8	Il18rap/Arrb2/Havcr2/Pik3r6/Cadm1/Lag3/Sh2d1b1/Klrb1b
GO:0032735	positive regulation of interleukin-12 production	0.025322878	6	Cd36/Ager/Rel/Ccr7/Tlr2/Tnfsf9
GO:0002715	regulation of natural killer cell mediated immunity	0.026123245	8	Il18rap/Arrb2/Havcr2/Pik3r6/Cadm1/Lag3/Sh2d1b1/Klrb1b
GO:0045581	negative regulation of T cell differentiation	0.026290353	7	Ctla4/Zc3h12a/Hlx/Il4ra/Runx1/Foxp3/Runx3
GO:0002689	negative regulation of leukocyte chemotaxis	0.026549353	4	Grem1/Dusp1/Nbl1/Slamf8
GO:0045579	positive regulation of B cell differentiation	0.026549353	4	Mmp14/Prdm1/Nckap1l/Inpp5d
GO:0043373	CD4-positive, alpha-beta T cell lineage commitment	0.026549353	4	Il6/Tgfb1/Batf/Foxp3

**TABLE 4:** Effects of Mesalazine on the DSS-altered cell signaling pathways.

KEGG ID	Description	Adjusted *p*-value	Number of gene	Gene ID
mmu04512	ECM-receptor interaction	1.60E-10	30	Thbs1/Col4a1/Tnc/Tnxb/Col4a2/Itgb8/Lamc1/Lamb1/Itga7/Lama4/Itga5/Itgb7/Col6a2/Col6a1/Thbs3/Npnt/Itgb3/Col9a2/Thbs2/Reln/Itga9/Sv2a/Sv2b/Fn1/Col1a2/Col4a5/Tnr/Hmmr/Itga8/Frem1
mmu04510	Focal adhesion	4.00E-08	43	Thbs1/Col4a1/Tnc/Tnxb/Col4a2/Itgb8/Lamc1/Lamb1/Myl9/Itga7/Lama4/Flna/Itga5/Itgb7/Col6a2/Col6a1/Pdgfra/Thbs3/Ppp1r12b/Pgf/Pdgfrb/Flt1/Pak3/Itgb3/Col9a2/Pdgfb/Thbs2/Reln/Shc4/Pak6/Itga9/Mapk10/Kdr/Shc2/Fn1/Pdgfc/Col1a2/Col4a5/Flt4/Tnr/Hgf/Itga8/Igf1
mmu04060	Cytokine-cytokine receptor interaction	4.67E-08	54	Ifng/Ifnl2/Cxcl9/Il1r1/Csf3/Cxcl1/Inhbb/Ifnl3/Il12rb2/Cxcr6/Cxcl11/Cxcl2/Bmp3/Ccl11/Bmp7/Ackr3/Il12rb1/Il5ra/Tnfsf11/Ppbp/Cxcl5/Cxcl3/Il6st/Il11/Il33/Il2rb/Pf4/Ccl21a/Tgfb3/Cd27/Ngfr/Il13ra2/Ccr7/Bmp6/Tnfrsf11b/Ackr4/Cxcr2/Cxcr3/Ccl22/Fasl/Il1rl1/Il6/Tgfb2/Tnfrsf12a/Il17d/Ccl7/Ccl2/Gm10591/Ccl5/Gm13304/Il19/Gdf10/Lepr/Eda
mmu04974	Protein digestion and absorption	6.55E-08	29	Col4a1/Col18a1/Col4a2/Col15a1/Col23a1/Col5a3/Col5a2/Col8a1/Col12a1/Col16a1/Slc36a4/Col6a2/Atp1b2/Col6a1/Col5a1/Col13a1/Col3a1/Gm2663/Col9a2/Col27a1/Slc8a2/Col11a1/Atp1a2/Eln/Col28a1/Col1a2/Col4a5/Col24a1/Col20a1
mmu04514	Cell adhesion molecules	9.44E-08	38	H2-DMb1/H2-DMa/H2-Aa/H2-Ab1/H2-Eb1/H2-Q7/Cd8a/Spn/Selp/Cd8b1/Itgb8/Nlgn2/H2-Q6/Itgb7/Cntnap2/Itgb2/Ntng1/Ncam1/Nrxn2/Cntn1/Vcan/Jam3/Cadm3/Cdh2/Nlgn1/Itga9/Ptprm/Negr1/Jam2/Cdh5/Esam/Cd2/Lrrc4b/Nrxn1/Nfasc/Cd28/Itga8/Cdh3
mmu04061	Viral protein interaction with cytokine and cytokine receptor	8.66E-07	25	Cxcl9/Cxcl1/Cxcl11/Cxcl2/Ccl11/Ackr3/Ppbp/Cxcl5/Cxcl3/Il6st/Il2rb/Pf4/Ccl21a/Ccr7/Ackr4/Cxcr2/Cxcr3/Ccl22/Il6/Ccl7/Ccl2/Gm10591/Ccl5/Gm13304/Il19
mmu04151	PI3K-Akt signaling pathway	8.66E-07	58	Thbs1/Col4a1/Ereg/Csf3/Tnc/Tnxb/Fgf7/Fgfr1/Col4a2/Itgb8/Lamc1/Lamb1/Itga7/Igf2/Lama4/Itga5/Itgb7/Col6a2/Col6a1/Pdgfra/Ppp2r2b/Thbs3/Gng4/Angpt2/Angpt1/Pgf/Nr4a1/Pdgfrb/Gng11/Gng3/Il2rb/Flt1/Efna5/Itgb3/Col9a2/Pdgfb/Ngfr/Thbs2/Reln/Itga9/Fasl/Ppp2r3a/Il6/Kdr/Fn1/Pdgfc/Ntrk2/Sgk1/Col1a2/Fgf10/Col4a5/Flt4/Tnr/Hgf/Ppp2r2c/Itga8/Igf1/Gm2436
mmu05323	Rheumatoid arthritis	2.39E-06	23	H2-DMb1/H2-DMa/H2-Aa/Ifng/H2-Ab1/Mmp3/H2-Eb1/Cxcl1/Cxcl2/Ctsk/Itgb2/Tnfsf11/Cxcl5/Angpt1/Cxcl3/Il11/Flt1/Tgfb3/Il6/Tgfb2/Ccl2/Ccl5/Cd28
mmu05144	Malaria	2.89E-06	18	Hba-a2/Hbb-bt/Hbb-bs/Hba-a1/Ifng/Thbs1/Csf3/Selp/Thbs3/Itgb2/Tgfb3/Gypc/Thbs2/Il6/Tgfb2/Ccl2/Klrk1/Hgf
mmu04657	IL-17 signaling pathway	1.97E-05	22	Ifng/S100a9/S100a8/Mmp3/Csf3/Cxcl1/Casp3/Cxcl2/Mmp9/Ccl11/Mmp13/Cxcl5/Cxcl3/Mapk4/Lcn2/Cebpb/Mapk10/Il6/Il17d/Ccl7/Ccl2/Ptgs2
mmu04020	Calcium signaling pathway	3.85E-05	40	Fgf7/Fgfr1/Nos1/Cacna1h/Ryr2/Pde1c/Bdkrb1/Ednra/Atp2b4/Bdkrb2/Pdgfra/Sphk1/Gdnf/Atp2b3/Pdgfrb/Pde1a/Ryr3/Ednrb/Flt1/Tacr1/Itpkb/Agtr1a/Pdgfb/Cacna1c/Slc8a2/Htr2b/Pln/Ret/Kdr/Ntrk3/Cckar/Pdgfc/Ntrk2/Fgf10/Casq2/Flt4/Ptgfr/P2rx2/Hgf/Avpr1a
mmu04726	Serotonergic synapse	9.49E-05	26	Adcy5/Dusp1/Ptgs1/Casp3/Cyp2d26/Gng4/Gabrb3/Htr3a/Gng11/Cyp2c66/Gng3/Alox5/Gnao1/Cyp2c68/Cacna1c/Gnai1/Alox15/Htr2b/Cyp2c55/Kcnj5/Slc18a2/Htr3b/Cyp2c65/Ptgs2/Trpc1/Kcnj3
mmu04080	Neuroactive ligand-receptor interaction	0.000330284	50	Sst/Gal/Penk/Grin3a/Grik3/Npy2r/Bdkrb1/Ednra/Chrna3/Bdkrb2/Calcb/Chrnb4/Gabrg3/Mtnr1a/Cnr1/Gzma/Pdyn/Gabrb3/Insl3/Calcrl/Adora1/Ptgir/Vip/Ednrb/Gm2663/Grm7/Tacr1/Grp/S1pr3/Agtr1a/Pth1r/Oprd1/Oprl1/Grik1/Htr2b/S1pr1/Glrb/Adra2a/Gria1/Mchr1/Cckar/Gabra3/Adcyap1r1/Grik5/Ptgfr/P2rx2/Agt/Lepr/Avpr1a/Sstr1
mmu04010	MAPK signaling pathway	0.000387987	43	Il1r1/Ereg/Cacna2d1/Dusp1/Fgf7/Fgfr1/Cacna1h/Hspa2/Igf2/Casp3/Flna/Mapk8ip2/Cacnb2/Pdgfra/Mapk8ip1/Angpt2/Angpt1/Pgf/Nr4a1/Ptpn5/Pdgfrb/Rasgrf2/Rasgrp1/Flt1/Tgfb3/Efna5/Map3k8/Ptpn7/Pdgfb/Ngfr/Cacna1c/Hspb1/Fasl/Mapk10/Tgfb2/Kdr/Pdgfc/Ntrk2/Fgf10/Cacng7/Flt4/Hgf/Igf1
mmu04658	Th1 and Th2 cell differentiation	0.000415763	19	H2-DMb1/H2-DMa/H2-Aa/Ifng/H2-Ab1/H2-Eb1/Tbx21/Il12rb2/Cd3g/Lck/Il12rb1/Cd247/Lat/Cd3d/Maf/Il2rb/Cd3e/Mapk10/Jag2
mmu04659	Th17 cell differentiation	0.000437294	21	H2-DMb1/H2-DMa/H2-Aa/Ifng/H2-Ab1/Il1r1/H2-Eb1/Tbx21/Cd3g/Lck/Il12rb1/Cd247/Lat/Cd3d/Il6st/Il2rb/Cd3e/Rora/Mapk10/Il6/Il17d
mmu04713	Circadian entrainment	0.000510163	20	Adcy5/Nos1/Cacna1h/Ryr2/Gucy1b1/Prkg1/Gng4/Mtnr1a/Gucy1a1/Gng11/Ryr3/Gng3/Gnao1/Cacna1c/Gnai1/Gria1/Kcnj5/Adcyap1r1/Prkg2/Kcnj3
mmu04360	Axon guidance	0.000510163	30	Myl9/Slit3/Nfatc4/Bmp7/Ntn4/Trpc4/Sema3a/Ntng1/Rgma/Pard6g/Efna5/Pak3/Epha5/Wnt5a/Enah/Gnai1/Pak6/Srgap3/Slit2/Ntn1/Sema3f/Trpc1/Ablim3/Ephb6/Unc5c/Epha7/Sema3d/Trpc3/Wnt4/Plxna4
mmu05414	Dilated cardiomyopathy	0.0008335	19	Adcy5/Cacna2d1/Itgb8/Ryr2/Itga7/Cacnb2/Itga5/Itgb7/Tgfb3/Itgb3/Cacna1c/Slc8a2/Itga9/Tgfb2/Pln/Cacng7/Agt/Itga8/Igf1
mmu05145	Toxoplasmosis	0.0008335	21	H2-DMb1/H2-DMa/H2-Aa/Ifng/Ciita/H2-Ab1/H2-Eb1/Lamc1/Lamb1/Hspa2/Casp3/Lama4/Socs1/Igtp/Tgfb3/Alox5/Gnao1/Gnai1/Irgm2/Mapk10/Tgfb2
mmu05146	Amoebiasis	0.001463118	20	Ifng/Col4a1/Il1r1/Cxcl1/Col4a2/Lamc1/Lamb1/Casp3/Lama4/Cxcl2/Itgb2/Cxcl3/Col3a1/Tgfb3/Hspb1/Il6/Tgfb2/Fn1/Col1a2/Col4a5
mmu05032	Morphine addiction	0.001463118	18	Adcy5/Pde1c/Gng4/Gabrg3/Gabrb3/Adora1/Grk3/Pde1a/Gng11/Gng3/Gnao1/Gnai1/Pde7b/Kcnj5/Gabra3/Pde8a/Pde4b/Kcnj3
mmu05410	Hypertrophic cardiomyopathy	0.001463118	18	Cacna2d1/Itgb8/Ryr2/Itga7/Cacnb2/Itga5/Itgb7/Tgfb3/Itgb3/Cacna1c/Slc8a2/Itga9/Il6/Tgfb2/Cacng7/Agt/Itga8/Igf1
mmu04014	Ras signaling pathway	0.001541131	34	Fgf7/Fgfr1/Igf2/Pla2g3/Pdgfra/Gng4/Angpt2/Lat/Angpt1/Pgf/Pdgfrb/Rasgrf2/Gng11/Rasgrp1/Gng3/Flt1/Efna5/Pak3/Pdgfb/Ngfr/Shc4/Pak6/Fasl/Mapk10/Pla1a/Kdr/Shc2/Pdgfc/Ntrk2/Fgf10/Flt4/Hgf/Pla2g2f/Igf1
mmu05321	Inflammatory bowel disease	0.001644514	14	H2-DMb1/H2-DMa/H2-Aa/Ifng/H2-Ab1/H2-Eb1/Tbx21/Il12rb2/Il12rb1/Maf/Tgfb3/Rora/Il6/Tgfb2
mmu05412	Arrhythmogenic right ventricular cardiomyopathy	0.001644514	16	Gja1/Cacna2d1/Itgb8/Ryr2/Itga7/Cacnb2/Itga5/Itgb7/Ctnna2/Itgb3/Cdh2/Cacna1c/Slc8a2/Itga9/Cacng7/Itga8
mmu04933	AGE-RAGE signaling pathway in diabetic complications	0.001648002	19	Col4a1/Mmp2/Serpine1/Col4a2/Casp3/Col3a1/Nox1/Tgfb3/Agtr1a/Thbd/Egr1/Mapk10/Il6/Tgfb2/Ccl2/Fn1/Col1a2/Col4a5/Agt
mmu04940	Type I diabetes mellitus	0.001666886	15	H2-DMb1/H2-DMa/H2-Aa/Ifng/Gzmb/Cpe/H2-Ab1/Prf1/H2-Eb1/H2-Q7/Ptprn/H2-Q6/Fasl/Gad2/Cd28
mmu05332	Graft-versus-host disease	0.001666886	14	H2-DMb1/H2-DMa/H2-Aa/Ifng/Gzmb/H2-Ab1/Prf1/H2-Eb1/H2-Q7/H2-Q6/Klrc1/Fasl/Il6/Cd28
mmu00590	Arachidonic acid metabolism	0.001666886	17	Ptgs1/Pla2g3/Ggt1/Cyp2c66/Alox5/Ptges/Cyp2c68/Cyp2e1/Ltc4s/Gpx3/Alox15/Cyp2c55/Ggt5/Cyp2c65/Ptgs2/Pla2g2f/Ptgis
mmu04640	Hematopoietic cell lineage	0.001666886	18	H2-DMb1/H2-DMa/H2-Aa/H2-Ab1/Il1r1/Csf3/H2-Eb1/Cd8a/Cd8b1/Cd3g/Itga5/Il5ra/Cd3d/Il11/Itgb3/Cd3e/Il6/Cd2
mmu04022	cGMP-PKG signaling pathway	0.001928048	27	Rgs2/Kcnj8/Adcy5/Myl9/Gucy1b1/Prkg1/Ednra/Atp2b4/Nfatc4/Atp1b2/Bdkrb2/Mrvi1/Kcnmb4/Atp2b3/Npr2/Gucy1a1/Adora1/Ednrb/Agtr1a/Oprd1/Cacna1c/Gnai1/Slc8a2/Atp1a2/Adra2a/Pln/Prkg2
mmu04062	Chemokine signaling pathway	0.001994739	29	Cxcl9/Adcy5/Cxcl1/Cxcr6/Cxcl11/Cxcl2/Ccl11/Gng4/Ppbp/Cxcl5/Cxcl3/Grk3/Gng11/Gng3/Pf4/Ccl21a/Itk/Ccr7/Gnai1/Shc4/Cxcr2/Cxcr3/Ccl22/Ccl7/Ccl2/Shc2/Gm10591/Ccl5/Gm13304
mmu04270	Vascular smooth muscle contraction	0.003271842	23	Adcy5/Myl9/Gucy1b1/Prkg1/Myh11/Ednra/Pla2g3/Calcb/Mrvi1/Ppp1r12b/Ramp3/Kcnmb4/Cald1/Npr2/Gucy1a1/Calcrl/Ptgir/Agtr1a/Cacna1c/Agt/Pla2g2f/Myh10/Avpr1a
mmu05143	African trypanosomiasis	0.003357978	10	Hba-a2/Hbb-bt/Hbb-bs/Hba-a1/Ido1/Ifng/Lama4/Ido2/Fasl/Il6
mmu04924	Renin secretion	0.003357978	15	Adcy5/Aqp1/Gucy1b1/Pde1c/Ednra/Clca1/Gucy1a1/Adora1/Pde1a/Agtr1a/Cacna1c/Gnai1/Adcyap1r1/Prkg2/Agt
mmu05142	Chagas disease	0.004334336	18	Ifng/Serpine1/Cd3g/Bdkrb2/Ppp2r2b/Cd247/Cd3d/Tgfb3/Gnao1/Cd3e/Gnai1/Fasl/Mapk10/Il6/Tgfb2/Ccl2/Ccl5/Ppp2r2c
mmu05165	Human papillomavirus infection	0.004334336	45	Thbs1/Col4a1/Tnc/H2-Q7/Tnxb/Col4a2/Itgb8/Lamc1/Lamb1/Itga7/Casp3/Lama4/H2-Q6/Itga5/Itgb7/Col6a2/Col6a1/Ppp2r2b/Thbs3/Hes2/Fzd1/Mx2/Pdgfrb/Pard6g/Itgb3/Col9a2/Wnt5a/Thbs2/Reln/Itga9/Wnt11/Fasl/Ppp2r3a/Apc2/Irf1/Fn1/Dlg2/Col1a2/Ptgs2/Col4a5/Tnr/Ppp2r2c/Itga8/Wnt4/Gm2436
mmu04390	Hippo signaling pathway	0.00461878	24	Serpine1/Nkd1/Wwtr1/Frmd6/Bmp7/Ppp2r2b/Itgb2/Lats2/Nkd2/Fzd1/Dlg4/Pard6g/Ctnna2/Tgfb3/Wnt5a/Bmp6/Wnt11/Tgfb2/Apc2/Dlg2/Gli2/Ppp2r2c/Sox2/Wnt4
mmu04015	Rap1 signaling pathway	0.004706209	30	Thbs1/Adcy5/Fgf7/Fgfr1/Pfn2/Pdgfra/Itgb2/Angpt2/Cnr1/Lat/Angpt1/Pgf/Pdgfrb/Pard6g/Flt1/Efna5/Gnao1/Itgb3/Pdgfb/Ngfr/Enah/Gnai1/Rapgef4/Kdr/Pdgfc/Fgf10/Flt4/Hgf/Skap1/Igf1
mmu05205	Proteoglycans in cancer	0.004814656	29	Thbs1/Mmp2/Fgfr1/Dcn/Hpse2/Twist2/Igf2/Casp3/Gpc3/Mmp9/Flna/Itga5/Ppp1r12b/Fzd1/Twist1/Itgb3/Lum/Wnt5a/Wnt11/Fasl/Tgfb2/Ank2/Kdr/Fn1/Col1a2/Hgf/Hoxd10/Wnt4/Igf1
mmu04612	Antigen processing and presentation	0.006298916	16	Cd74/H2-DMb1/H2-DMa/H2-Aa/Ifng/Ciita/H2-Ab1/H2-Eb1/H2-Q7/Cd8a/Lgmn/Cd8b1/Hspa2/Tap1/H2-Q6/Klrc1
mmu04926	Relaxin signaling pathway	0.009364211	20	Col4a1/Adcy5/Mmp2/Nos1/Col4a2/Mmp9/Mmp13/Gng4/Insl3/Gng11/Col3a1/Ednrb/Gng3/Gnao1/Gnai1/Shc4/Mapk10/Shc2/Col1a2/Col4a5
mmu04725	Cholinergic synapse	0.010129103	18	Adcy5/Slc18a3/Chrna3/Kcnq4/Kcnq3/Kcnj12/Chrnb4/Gng4/Chat/Gng11/Gng3/Gnao1/Cacna1c/Gnai1/Kcnq2/Slc5a7/Kcnq5/Kcnj3
mmu04350	TGF-beta signaling pathway	0.010577012	16	Ifng/Thbs1/Nbl1/Inhbb/Dcn/Fst/Ltbp1/Grem1/Grem2/Bmp7/Rgma/Fbn1/Tgfb3/Pitx2/Bmp6/Tgfb2
mmu04724	Glutamatergic synapse	0.010726962	18	Adcy5/Grin3a/Grik3/Gng4/Dlg4/Grk3/Gng11/Gng3/Shank1/Grm7/Gnao1/Cacna1c/Gnai1/Grik1/Gria1/Grik5/Trpc1/Kcnj3
mmu05330	Allograft rejection	0.012447412	12	H2-DMb1/H2-DMa/H2-Aa/Ifng/Gzmb/H2-Ab1/Prf1/H2-Eb1/H2-Q7/H2-Q6/Fasl/Cd28
mmu04261	Adrenergic signaling in cardiomyocytes	0.012463153	22	Adcy5/Cacna2d1/Ryr2/Scn7a/Atp2b4/Cacnb2/Atp1b2/Ppp2r2b/Atp2b3/Scn5a/Agtr1a/Cacna1c/Gnai1/Slc8a2/Atp1a2/Ppp2r3a/Rapgef4/Pln/Cacng7/Agt/Ppp2r2c/Gm2436
mmu00591	Linoleic acid metabolism	0.018463113	10	Cyp3a44/Cyp3a25/Pla2g3/Cyp2c66/Cyp2c68/Cyp2e1/Alox15/Cyp2c55/Cyp2c65/Pla2g2f
mmu04610	Complement and coagulation cascades	0.01983992	15	Serpine1/Bdkrb1/Fga/Bdkrb2/Itgb2/Pros1/Thbd/Tfpi/C6/F10/C7/Plat/C2/Fgg/F13a1
mmu04660	T cell receptor signaling pathway	0.02148628	16	Ifng/Cd8a/Cd8b1/Cd3g/Lck/Cd247/Lat/Cd3d/Rasgrp1/Pak3/Map3k8/Itk/Cd3e/Pak6/Mapk10/Cd28
mmu04810	Regulation of actin cytoskeleton	0.022111954	28	Fgf7/Fgfr1/Itgb8/Myl9/Itga7/Myh11/Bdkrb1/Itga5/Itgb7/Pfn2/Bdkrb2/Pdgfra/Ppp1r12b/Itgb2/Pdgfrb/Pak3/Itgb3/Pdgfb/Enah/Cyfip2/Pak6/Itga9/Apc2/Fn1/Pdgfc/Fgf10/Myh10/Itga8
mmu04668	TNF signaling pathway	0.022320614	17	Mmp3/Cxcl1/Casp3/Cxcl2/Mmp9/Socs3/Cxcl5/Cxcl3/Cebpb/Map3k8/Mapk10/Il6/Irf1/Ccl2/Ccl5/Ifi47/Ptgs2
mmu04540	Gap junction	0.022406629	14	Adcy5/Gja1/Gucy1b1/Prkg1/Pdgfra/Tubb3/Gucy1a1/Pdgfrb/Tubb4a/Pdgfb/Gnai1/Htr2b/Pdgfc/Prkg2
mmu00603	Glycosphingolipid biosynthesis - globo and isoglobo series	0.022901215	5	St3gal1/B3galnt1/Fut9/Fut1/St3gal2
mmu04921	Oxytocin signaling pathway	0.024501045	21	Rgs2/Adcy5/Cacna2d1/Ryr2/Myl9/Gucy1b1/Nfatc4/Cacnb2/Ppp1r12b/Kcnj12/Npr2/Gucy1a1/Ryr3/Gnao1/Cacna1c/Gnai1/Kcnj5/Camkk2/Ptgs2/Cacng7/Kcnj3
mmu04978	Mineral absorption	0.024501045	10	Mt2/Atp2b4/Atp1b2/Slc26a3/Atp2b3/Mt1/Slc34a2/Slc8a2/Atp1a2/Trf
mmu04728	Dopaminergic synapse	0.026573382	19	Adcy5/Ppp2r2b/Kif5a/Gng4/Gng11/Gng3/Gnao1/Cacna1c/Gnai1/Mapk10/Ppp2r3a/Gria1/Kcnj5/Slc18a2/Kif5c/Kcnj3/Caly/Ppp2r2c/Gm2436
mmu04630	JAK-STAT signaling pathway	0.033932961	22	Ifng/Ifnl2/Csf3/Ifnl3/Gfap/Il12rb2/Socs1/Socs2/Pdgfra/Il12rb1/Il5ra/Socs3/Il6st/Il11/Pdgfrb/Il2rb/Pdgfb/Il13ra2/Il6/Il17d/Il19/Lepr
mmu05310	Asthma	0.037355741	6	H2-DMb1/H2-DMa/H2-Aa/H2-Ab1/H2-Eb1/Ccl11

### Immunosuppressive Effect of Mesalazine via the Regulation of Canonical Pathway

**FIGURE 3:**
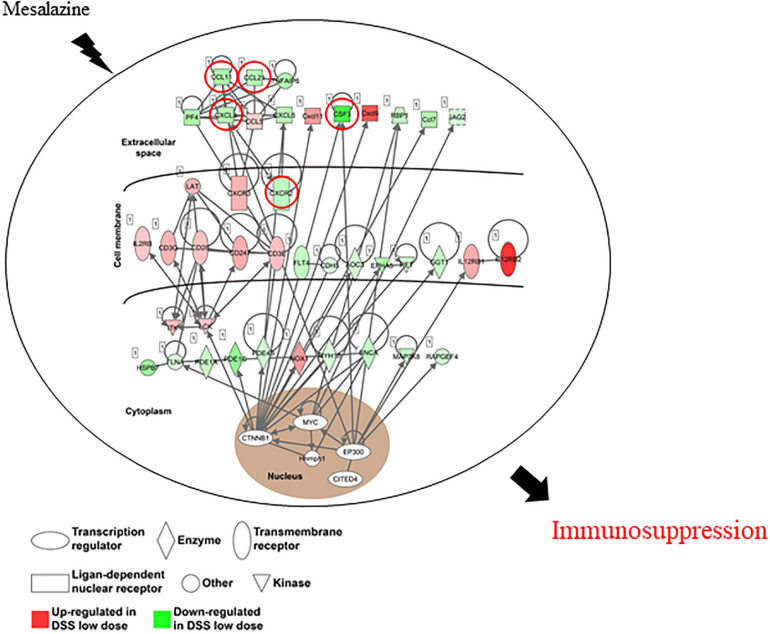
The gene network involved in the immunosuppressive effect of mesalazine on DSS-induced colitis. Ingenuity pathway analysis shows the contribution of different molecules in the immunosuppressive effect of mesalazine. The red symbol represents upregulated genes; the green shape represents downregulated genes.

The DEGs were then subjected to IPA to determine the immunosuppressive effect of mesalazine on the treatment of UC. The results of canonical pathway analysis highlighted the substantial alteration of a large number of cell signaling pathways related to immune and inflammatory responses (*p* < 0.05) (Table 5). They included the regulation of interleukin signaling, such as IL-6, IL-8, IL-15, and IL-17 signaling pathways (Table 5). Furthermore, the results highlighted T cell differentiation, including Th1, Th2, and T helper cells (Table 5). In addition to the T cells, we also observed the regulation of B cells by mesalazine treatment (Table 5). Finally, we conducted IPA gene network analysis to delineate the detailed molecular mechanisms underlying the immunosuppressive responses caused by mesalazine. Our results showed that mesalazine regulated a large number of C–C motif chemokine ligands (CCL5, CCL7, CCL11, and CL21) and C-X-C motif chemokine ligands (CXCL3, CXCL6, CXCL9, and CXCL11) in the extracellular space (Figure 3). This led to the modulation of a group of transmembrane receptors, including interleukin receptors (IL2RB, IL12RB1, and IL12RB2), T-cell surface glycoprotein (CD3E, CD3G, CD28, and CD247), and chemokine receptors (CXCR2 and CXCR3) (Figure 3). The regulation of these receptors has been reported to regulate different enzymes (PDE1A, PDE1B, PDE1C, and MYH10) and kinases (ITK, LCK NOX1, and MAP3K8) involved in immune responses (Figure 3). More importantly, mesalazine treatment reversed the suppressive effect of immune responsive genes, including chemokine (C–X–C motif) ligand (CXCL3), chemokine (C–C motif) ligand (CCL11 and CCL21), chemokine receptor (CXCR2), and colony-stimulating factor (CSF3) in the DSS-induced colitis model (Table 6). Taken together, our results suggest that mesalazine treatment could reverse the DSS-induced immune response through its immunosuppressive effect.

**TABLE 5:** Effects of Mesalazine on the IPA canonical pathways.

Ingenuity Canonical Pathways	Adjusted *p*-value	Molecules
Agranulocyte Adhesion and Diapedesis	4.46684E-09	AOC3,CCL11,Ccl2,CCL21,CCL22,CCL5,Ccl7,CDH5,Cxcl11, CXCL2,Cxcl3,CXCL3,CXCL6,Cxcl9,CXCR2,FN1,GNAI1,IL1R1, IL33,ITGA5,ITGB2,ITGB7,JAM3,MMP10,MMP13,MMP19, MMP2,MMP3,MMP7,MMP9,MYH10,MYH11,MYL9,PF4, PODXL2,Ppbp,SELP
Th1 and Th2 Activation Pathway	7.07946E-09	CD247,CD28,CD3D,CD3E,CD3G,CD8A,CXCR3,CXCR6,HLA-A, HLA-DMA,HLA-DMB,HLA-DQA1,HLA-DQB1,HLA-DRB5,IFNG,IKZF1, IL12RB1,IL12RB2,IL1RL1,IL2RB,IL33,IL6,IRF1,ITGB2,JAG2,KLRC1, MAF,NFATC4,S1PR1,SOCS1,SOCS3,TBX21,TGFBR3,TNFSF11
Th1 Pathway	3.16228E-07	CD247,CD28,CD3D,CD3E,CD3G,CD8A,CXCR3,HLA-A,HLA-DMA, HLA-DMB,HLA-DQA1,HLA-DQB1,HLA-DRB5,IFNG,IL12RB1,IL12RB2, IL6,IRF1,ITGB2,KLRC1,NFATC4,SOCS1,SOCS3,TBX21,TNFSF11
Th2 Pathway	8.91251E-07	CD247,CD28,CD3D,CD3E,CD3G,CXCR6,HLA-A,HLA-DMA,HLA-DMB, HLA-DQA1,HLA-DQB1,HLA-DRB5,IFNG, IKZF1,IL12RB1,IL12RB2,IL1RL1,IL2RB,IL33,ITGB2,JAG2,MAF, S1PR1,SOCS3,TBX21,TGFBR3
Antigen Presentation Pathway	5.12861E-06	CD74,CIITA,HLA-A,HLA-DMA,HLA-DMB,HLA-DQA1,HLA-DQB1, HLA-DRB5,IFNG,PSMB8,PSMB9,TAP1
T Helper Cell Differentiation	7.58578E-05	CD28,HLA-A,HLA-DMA,HLA-DMB,HLA-DQA1,HLA-DQB1, HLA-DRB5,IFNG,IL12RB1,IL12RB2,IL6,IL6ST,NGFR,TBX21,TNFRSF11B
Neuroinflammation Signaling Pathway	0.0001	CALB2,CASP3,CCL5,CD200,FASLG,FZD1,GABRA3,GABRB3, GABRG3,GAD2,GDNF,GRIA1,GRIN3A,HLA-A,HLA-DMA,HLA-DMB, HLA-DQA1,HLA-DQB1,HLA-DRB5,IFNG,IL1R1,IL6,KCNJ3,KCNJ5, KLK3,MAPK10,MAPK4,MMP3,MMP9,NFATC4,NOX1,PLA2G2F, PLA2G3,PTGS2,SNCA,TGFB2,TGFB3,TGFBR3
Cardiac Î^2^-adrenergic Signaling	0.000169824	ADCY5,AKAP12,AKAP6,CACNA1C,GNG11,GNG3,GNG4,GRK3, PDE1A,PDE1C,PDE4B,PDE7B,PDE8A,PKIB,PPP1R3C,PPP2R2B, PPP2R2C,PPP2R3A,PRKAR1B,RYR2,SLC8A2,SMPDL3B
IL-15 Production	0.000389045	AATK,AXL,DDR2,EPHA5,FGFR1,FLT1,FLT4,IL6,IRF1,ITK,KDR,LCK, NTRK2,NTRK3,PDGFRA,PDGFRB,RET,ROR2,TIE1
CCR5 Signaling in Macrophages	0.000436516	CACNA1C,CACNA1H,CACNA2D1,CACNB2,CACNG7,CCL5,CD247, CD3D,CD3E,CD3G,FASLG,GNAI1,GNG11,GNG3,GNG4,MAPK10
IL-17 Signaling	0.000660693	CCL11,CCL22,CEBPB,CSF3,CXCL3,Eda,FASLG,IFNG,IL11,IL17D, IL33,IL6,LCN2,MAPK10,MMP13,MMP2,MMP3,MMP9,PDGFC,PGF,PTGS2,TGFB2,TGFB3,TNFRSF11B,TNFSF11
iCOS-iCOSL Signaling in T Helper Cells	0.001023293	CD247,CD28,CD3D,CD3E,CD3G,HLA-A,HLA-DMA,HLA-DMB, HLA-DQA1,HLA-DQB1,HLA-DRB5,IL2RB,ITK,LAT,LCK, NFATC4,PLEKHA4
Leukocyte Extravasation Signaling	0.001047129	ARHGAP6,ARHGAP8/PRR5-ARHGAP8,CDH5,CTNNA2,DLC1,EDIL3, GNAI1,ITGB2,ITK,JAM2,JAM3,MAPK10,MMP10,MMP13,MMP19,MMP2,MMP3,MMP7,MMP9,NOX1,RAPGEF4,RASGRP1,SPN,TIMP1,TIMP2
Calcium-induced T Lymphocyte Apoptosis	0.001230269	CD247,CD3D,CD3E,CD3G,HLA-A,HLA-DMA,HLA-DMB, HLA-DQA1,HLA-DQB1,HLA-DRB5,LCK,NR4A1
Cytotoxic T Lymphocyte-mediated Apoptosis of Target Cells	0.001412538	CASP3,CD247,CD3D,CD3E,CD3G,FASLG,HLA-A,PRF1
ILK Signaling	0.001819701	CASP3,FERMT2,FLNA,FN1,ITGB2,ITGB3,ITGB7,ITGB8,LIMS2, MAPK10,MMP9,MYH10,MYH11,MYL9,PDGFC,PGF,PPP2R2B, PPP2R2C,PPP2R3A,PTGS2,RHOJ,RND2,SNAI1,TGFB1I1
Acute Phase Response Signaling	0.001949845	AGT,C2,CEBPB,CP,FGA,FGG,FN1,HP,IL1R1,IL33,IL6,IL6ST,NGFR,RBP1,SAA1,Saa3,SERPINA3,SERPINE1,SOCS1,SOCS2,SOCS3,TF,TNFRSF11B
Interferon Signaling	0.002089296	IFIT1,IFIT3,IFITM2,IFNG,IRF1,PSMB8,SOCS1,TAP1
PKCÎ^2^ Signaling in T Lymphocytes	0.003311311	CACNA1C,CACNA1H,CACNA2D1,CACNB2,CACNG7,CD247,CD28, CD3D,CD3E,CD3G,HLA-A,HLA-DMA,HLA-DMB,HLA-DQA1, HLA-DQB1,HLA-DRB5,LAT,LCK,MAP3K8,NFATC4
Autoimmune Thyroid Disease Signaling	0.004466836	CD28,FASLG,HLA-A,HLA-DMA,HLA-DMB,HLA-DQA1,HLA-DQB1, HLA-DRB5,PRF1
CTLA4 Signaling in Cytotoxic T Lymphocytes	0.005754399	CD247,CD28,CD3D,CD3E,CD3G,CD8A,CD8B,HLA-A,LAT,LCK, PPP2R2B,PPP2R2C,PPP2R3A
CD28 Signaling in T Helper Cells	0.006309573	CD247,CD28,CD3D,CD3E,CD3G,HLA-A,HLA-DMA,HLA-DMB, HLA-DQA1,HLA-DQB1,HLA-DRB5,ITK,LAT,LCK,MAPK10,NFATC4
Altered T Cell and B Cell Signaling in Rheumatoid Arthritis	0.006309573	CCL21,CD28,FASLG,HLA-A,HLA-DMA,HLA-DMB,HLA-DQA1, HLA-DQB1,HLA-DRB5,IFNG,IL33,IL6,TNFSF11
B Cell Development	0.00851138	HLA-A,HLA-DMA,HLA-DMB,HLA-DQA1,HLA-DQB1, HLA-DRB5,SPN
Role of IL-17A in Psoriasis	0.011220185	CXCL3,CXCL6,S100A8,S100A9
Leukotriene Biosynthesis	0.011220185	ALOX5,GGT1,GGT5,LTC4S
Antiproliferative Role of TOB in T Cell Signaling	0.013182567	CD247,CD28,CD3D,CD3E,CD3G,TGFB2,TGFB3
IL-8 Signaling	0.013803843	ANGPT1,ANGPT2,CXCR2,FLT1,FLT4,GNAI1,GNG11,GNG3, GNG4,ITGB2,ITGB3,KDR,MAPK10,MMP2,MMP9,MYL9,NOX1,PDGFC,PGF,PTGS2,RHOJ,RND2
Role of NFAT in Regulation of the Immune Response	0.017378008	CD247,CD28,CD3D,CD3E,CD3G,GNAI1,GNAO1,GNG11,GNG3, GNG4,HLA-A,HLA-DMA,HLA-DMB,HLA-DQA1,HLA-DQB1, HLA-DRB5,ITK,LAT,LCK,NFATC4
Role of JAK family kinases in IL-6-type Cytokine Signaling	0.022387211	IL6,IL6ST,MAPK10,SOCS1,SOCS3
T Cell Receptor Signaling	0.023442288	CD247,CD28,CD3D,CD3E,CD3G,CD8A,CD8B,ITK,LAT,LCK, NFATC4,PTPN7,RASGRP1
T Cell Exhaustion Signaling Pathway	0.023442288	CD28,HLA-A,HLA-DMA,HLA-DMB,HLA-DQA1,HLA-DQB1,HLA-DRB5,IFNG,IL12RB1,IL12RB2,IL6,KDR,MAPK10,NFATC4,PPP2R2B, PPP2R2C,PPP2R3A,TBX21,TGFBR3
Nur77 Signaling in T Lymphocytes	0.035481339	CASP3,CD247,CD28,CD3D,CD3E,CD3G,FASLG,HDAC9,NR4A1
IL-6 Signaling	0.039810717	CEBPB,HSPB1,HSPB7,IL1R1,IL1RL1,IL33,IL6,IL6ST,MAPK10,NGFR, SOCS1,SOCS3,TNFAIP6,TNFRSF11B

**TABLE 6:** Reversed effects of Mesalazine on the DSS-altered genes.

Sample	Valid reads	Mapped reads
Ctrl_1	51703938	49304169(95.36%)
Ctrl_2	48764866	46876854(96.13%)
Ctrl_3	54347766	49174003(90.48%)
Ctrl_4	49696000	47928769(96.44%)
Ctrl_5	46060298	44134098(95.82%)
Dss_1	50340262	48027779(95.41%)
Dss_2	50620446	48256329(95.33%)
Dss_3	49932850	47515351(95.16%)
Dss_4	50142292	47646669(95.02%)
Dss_5	50198506	48609721(96.83%)
Dss + Mesalazine_1	48853522	47338989(96.90%)
Dss + Mesalazine_2	46903500	45351455(96.69%)
Dss + Mesalazine_3	46894200	45413947(96.84%)
Dss + Mesalazine_4	43217994	41856731(96.85%)
Dss + Mesalazine_5	46387314	44981533(96.97%)

In the later part of this study, we aimed to determine the molecular pathways underlying the immunosuppressive effect of mesalazine on DSS-induced colitis. Therefore, we focused on immune-related biological processes and pathways in bioinformatic analysis. Our results show that mesalazine treatment can alter different immune cells, including T cells, B cells, and natural killer cells, leading to the control of autoimmune responses (Zitti and Bryceson, 2018; Meffre and O’Connor, 2019; Wing et al., 2019). T cells are reported to contribute to the pathogenesis of chronic intestinal inflammation and are considered targets for UC treatment due to their role in the autoimmune system (Eri et al., 2012). For example, a study using a T cell transfer colitis model demonstrated that CD4(+) T helper cells strongly contributed to the pathogenesis of IBD (Maschmeyer et al., 2021). In addition, imbalanced CD4+ T cells have been reported as predisposing factors for colitis (Pandit et al., 2021). Furthermore, mesalazine treatment mediated cytokine biosynthesis and secretion. As mentioned before, cytokines play an important role in the autoimmune response. More importantly, our gene network analysis further highlighted the suppression of C–C motif chemokine ligands (CCL11 and CCL21) and C–X–C motif chemokine ligands (CXCL3 and CXCR2) by mesalazine treatment. All of these ligands were increased in the DSS-induced model. CCL11, a prototypical Th2 chemokine, is associated with enrichment in Th2 CD4+ T cells (Chao et al., 2014) and is involved in eosinophil recruitment (Hornig et al., 2016). CCL11 expression levels correlated with the expression levels of different ILs. Reduced expression of CCL11 has been reported to alleviate allergic symptoms and allergic inflammatory responses by reducing serum cytokine levels (Lei et al., 2020). It has been reported that CD4+ CD25+ T cells inhibit the development of colitis induced by both Th1 and Th2 cells in a mouse model (Xu et al., 2003). The Th1/Th2 balance of peripheral CD4-positive T cells is considered a biomarker for patients with refractory ulcerative colitis (Nakase et al., 2009). CCL21, a ligand of the receptor CCR7, contributes to the balance of immunity and tolerance through thymocyte development, secondary lymphoid organogenesis, high affinity antibody responses, and regulatory and memory T-cell function (Förster et al., 2008; Comerford et al., 2013). It has been shown that the loss of CCL21 responsiveness in the normal development of the memory T-cell effector function does not hold for autoimmune diseases (Christopherson et al., 2003). A mouse colitis study demonstrated that CCL21 suppression could decrease damage induced by ulcerative colitis (Zhang et al., 2014), suggesting that CCL21 might be a therapeutic target for UC treatment.

### Anti-colitis Actions of Mesalazine *in vivo*

**FIGURE 4:**
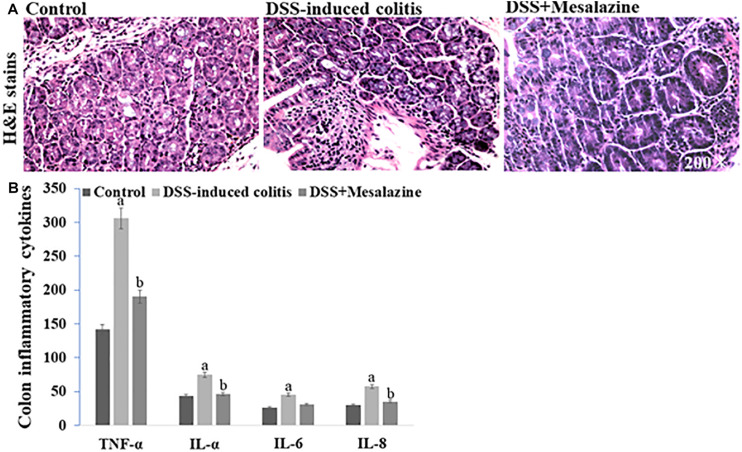
*In vivo* effects of mesalazine treatment on DSS-induced colitis. **(A)** Hematoxylin and eosin staining show the structural changes including visible crypt abscess, inflammatory infiltration, and cytoclasis in DSS-induced colitis. These alterations were reversed by mesalazine treatment. **(B)** Mesalazine treatment relieved DSS-induced inflammatory cytokines including TNF-α, IL-1α, IL-6, and IL-8 in the colon samples via its anti-inflammation action.

To determine the pharmacological effects of mesalazine against colitis, the structure of the colon tissues was studied. Our results showed that the length of the colon was shortened and colon tissues became fragile in the colitis mouse model, and ulcers were formed, including mucosal abscess and congestion. Mesalazine intervention improved pathological changes in mice with colitis. Histomorphological observations using H&E staining showed that the colon tissue of the colitis mice changed in morphology and showed alterations, such as crypt abscess, decreased density in the colon, and inflammatory infiltration. Mesalazine treatment mitigated mucosal structural changes and reduced inflammatory infiltration (Figure 4A). ELISA data showed that the expression levels of TNF-α, IL-1α, IL-6, and IL-8 in colitis mice were significantly reduced compared with those in control samples. Interestingly, the elevation of inflammatory cytokines in mesalazine-treated colitis mice was decreased (Figure 4B).

CXCL3, a small cytokine, controls the migration and adhesion of monocytes through its interaction with the cell surface chemokine receptor CXCR2 (Smith et al., 2005). CXCR2 is a functional receptor for GRO-family chemokines involved in monocyte recruitment and inhibits inflammation. CXCR2 has been found to be significantly increased in colonic mucosal tissues of patients with active UC (Zhu et al., 2020). It has been reported that CXCL3 plays a role in controlling intestinal inflammation and gut mucosal healing, and is considered a key prognostic parameter in the management of IBD (Stronati et al., 2019). Therefore, the suppressive effect of mesalazine on these C–C motif chemokine ligands and C–X–C motif chemokine ligands suggests a possible strategy for the treatment of UC, and a better understanding of the molecular mechanism underlying the immunosuppressive effect of mesalazine has important implications for developing novel anti-UC drugs directed against the CXC chemokine signaling system. However, the newly identified markers need to be further confirmed and investigated using a human model.

## Data Availability Statement

The data presented in the study are deposited in the BioProject repository, accession number PRJNA732984.

## Ethics Statement

The animal study was reviewed and approved by West China School of Basic Medical Sciences and Forensic Medicine of Sichuan University.

## Author Contributions

RL and LZ conceived and designed the experiments and wrote the manuscript. RL, LC, and QW performed the experiments. RL analysed the data. All authors read and approved the final manuscript.

## Conflict of Interest

The authors declare that the research was conducted in the absence of any commercial or financial relationships that could be construed as a potential conflict of interest.

## Publisher’s Note

All claims expressed in this article are solely those of the authors and do not necessarily represent those of their affiliated organizations, or those of the publisher, the editors and the reviewers. Any product that may be evaluated in this article, or claim that may be made by its manufacturer, is not guaranteed or endorsed by the publisher.

## Footnotes

1
http://genome.ucsc.edu/
